# The sensitivity of donor – acceptor charge transfer to molecular geometry in DAN – NDI based supramolecular flower-like self-assemblies

**DOI:** 10.1038/s41598-017-15599-9

**Published:** 2017-11-28

**Authors:** Mohammad Al Kobaisi, Rajesh S. Bhosale, Mohamed E. El-Khouly, Duong Duc La, Sachin D. Padghan, Sidhanath V. Bhosale, Lathe A. Jones, Frank Antolasic, Shunichi Fukuzumi, Sheshanath V. Bhosale

**Affiliations:** 1 School of Science, RMIT University, GPO Box 2476, Melbourne, VIC-3001 Australia; 2 Polymers and Functional Materials Division, CSIR-Indian Institute of Chemical Technology, Hyderabad, 500007 Telangana India; 3 Department of Chemistry, Faculty of Science, Kafrelsheikh University, Kafrelsheikh, 33516 Egypt; 4 Department of Chemistry and Nano Science, Ewha Womans University, Seoul, 03760 Korea; 5 Faculty of Science and Engineering, Meijo University, Nagoya, Aichi 468-8502 Japan

## Abstract

A charge-transfer (CT) complex self-assembled from an electron acceptor (NDI-EA: naphthalene diimide with appended diamine) and an electron donor (DAN: phosphonic acid-appended dialkoxynapthalene) in aqueous medium. The aromatic core of the NDI and the structure of DAN1 were designed to optimize the dispersive interactions (π-π and van der Waals interactions) in the DAN1–NDI-EA self-assembly, while the amino groups of NDI also interact with the phosphonic acid of DAN1 via electrostatic forces. This arrangement prevented crystallization and favored the directional growth of 3D flower nanostructures. This molecular geometry that is necessary for charge transfer to occur was further evidenced by using a mismatching DAN2 structure. The flower-shaped assembly was visualized by scanning electron and transmission electron microscopy. The formation of the CT complex was determined by UV-vis and cyclic voltammetry and the photoinduced electron transfer to produce the radical ion pair was examined by femtosecond laser transient absorption spectroscopic measurements.

## Introduction

The field of organic optoelectronics has expanded dramatically in recent years, where systems including organic light-emitting devices (OLEDs) and organic photovoltaic (OPV) devices have shown great promise and rapid advances^1^. It is essential to understand the structure–properties relationships of these systems in order to design, optimize and control their photoelectronic functions. Such devices depend strongly on efficient charge separation in donor – acceptor systems. The association of donor and acceptor molecules often results in the modification of the electronic structure of the system and formation of charge transfer (CT) complexes^2^. CT complexes may further self-assemble to give crystalline or supramolecular structures. Such assemblies are capable of giving CT complexes, which result in partial or integer electron transfer from the donor to the acceptor part of the structure formed.

Naphthalene diimide (NDI) is a well-known versatile π-conjugated electron acceptor^3–10^, and dialkoxynaphthalene (DAN) is a relatively good electron donor^11^, which can be used to design and construct CT complexes^12–20^. Cubberley *et al*. demonstrated that the NDI and DAN association constant is changed by 1 to 2 orders of magnitude by the nature of the substitutions and the geometry of the assemblies^21^. Attaching ionisable moieties to the aromatic cores to increase the negative charge on the DAN and the positive charge on the NDI provides a strong electrostatic interaction in addition to the π-π interactions, which result in face-centred stacking of these two species. It has been shown that this pair in polymeric form can produce alternating superstructures in the form of foldamers^22,23^. Various molecular design strategies have been used to enhance the assembly and electronic interactions between NDI and DAN^11–23^. For example strong electron withdrawing moieties on the acceptor, such as fluorine, are combined with the substitution of strong electron donating moieties on the donor, such as thiophenes^24^.

This approach is used to design CT complexes with HOMO – LUMO gaps in the near infrared (NIR) and IR regions^25–27^. The simple NDI – DAN CT complexes studied here show HOMO – LUMO gaps just below 700 nm which can be further tuned to shift to lower energy.

**Figure 1:**
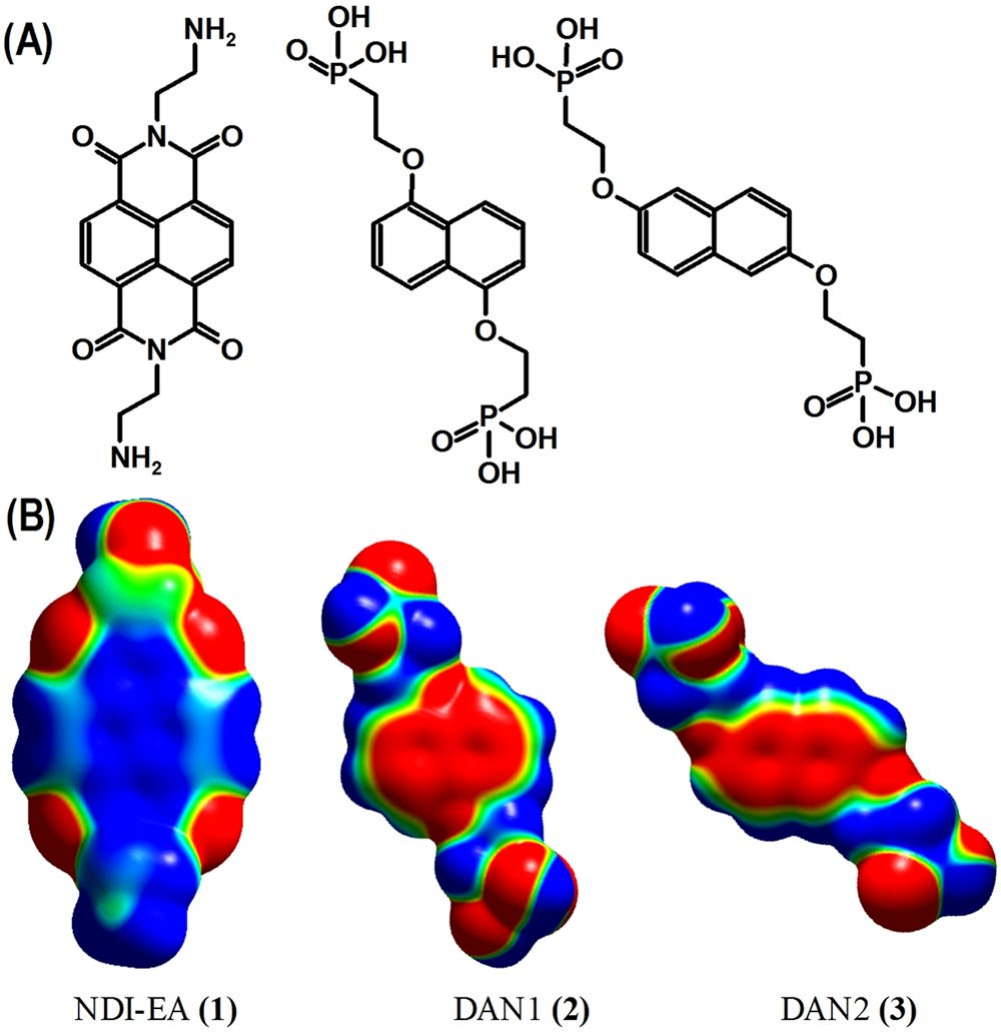
The chemical structure: *N*,*N*′-diethylamine-NDI and 1,5- and 2,7-di(1-ethoxy 2-phosphonate) i.e. DAN1 and DAN2, and their total electron density distribution as calculated using TD DFT (B3LYP/6-31 G + dp) level of theory, where the blue areas are electron deficient and the red areas are electron rich.

For electron transfer to occur in a NDI – DAN based foldamers or co-assembly; it requires an appropriate molecular conformation, Molecular Orbital (MO) symmetry and charge density distribution. In the self-assemblies studied here, we show the hybridisation between the MOs in the donor and acceptor molecules is ruled by the molecular geometry and orbital symmetry, which can result in an intricate overlap of charge densities of the two molecules in the complex to give a new set of frontier MOs and new optical and electronic features. We have also studied the effect of molecular conformation on the HOMO – LUMO transition in the NIR region in a NDI – DAN CT complex, in which both molecules carry ionic moieties. The acceptor is NDI with *N*,*N*′-ethylamine substitution (NDI-EA) **(1)** and the donors are 1,5- and 2,7-di(1-ethoxy 2-phosphonate) of DAN (DAN1 **(2)** and DAN2 **(3)** respectively) (Fig. 1). There is a strong interaction between the amine and phosphonate functional groups in their ionized states. Here we have also examined the effect of the co-assembly of NDI-EA – DAN1 and NDI-EA – DAN2 on the secondary supramolecular structure.

## Results

Synthesis of the *N*,*N*′-diethylamine-NDI **(1)** (NDI-EA) was undertaken following a literature protocol (ESI Scheme S1)^16^. The synthesis of compound 1,5- i.e. DAN1 **(2)**, 2,7-di(1-ethoxy 2-phosphonate) i.e. DAN2 **(3)** were achieved using a multistep synthetic strategy (ESI Scheme S2).

### Charge Transfer Complex

**Figure 2:**
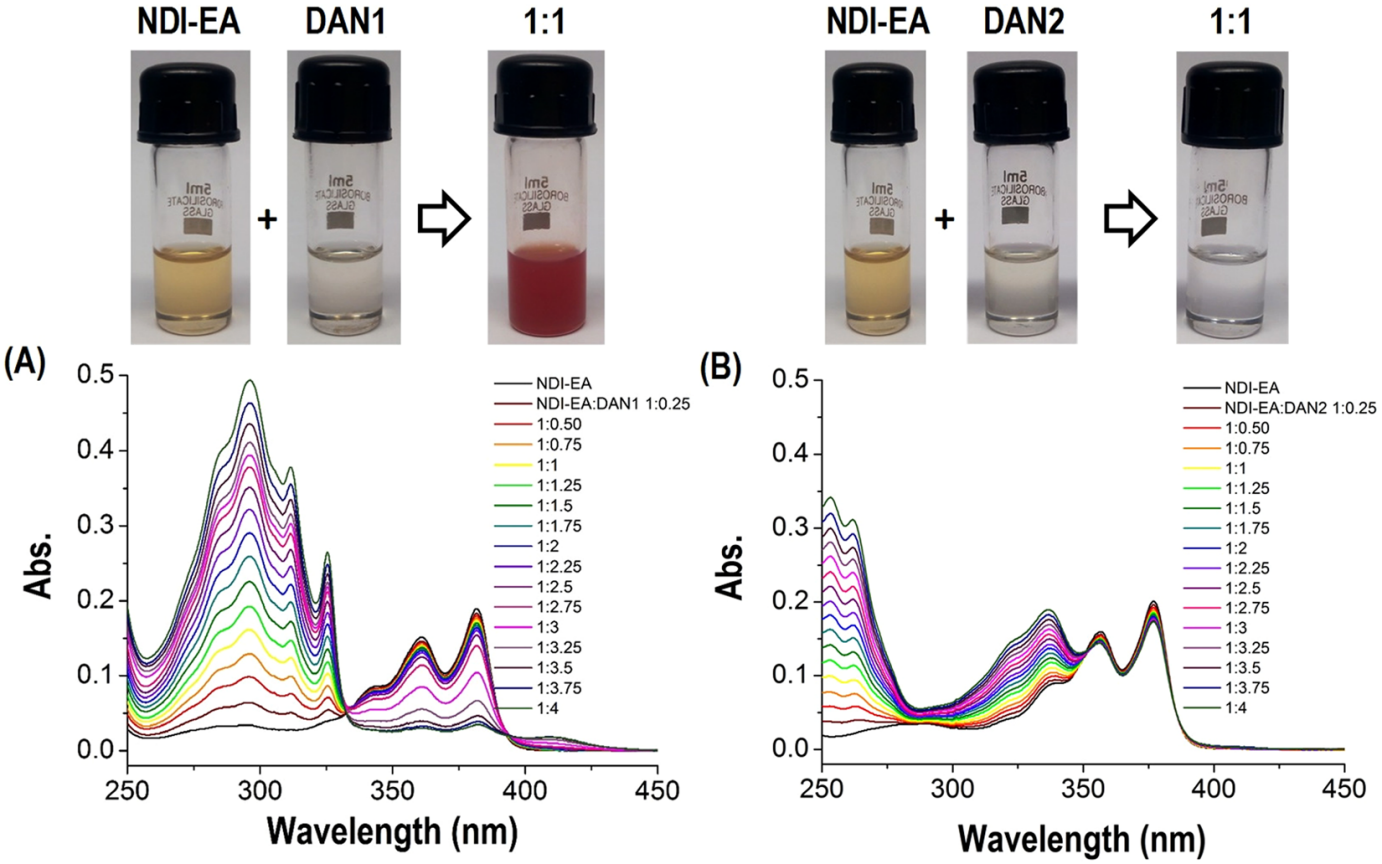
Charge Transfer Complex: The visual appearance of NDI-EA (**1**), DAN1 (**2**) and DAN2 (**3**) solutions in ultrapure water in their pure form, and the UV-vis spectra of NDI-EA with DAN1 (**A**), and DAN2 (**B**) at various ratios, and 1:1 molar ratio with visual appearance of the solutions.

The formation of NDI-EA – DAN1 (**Com1**) and NDI-EA – DAN2 (**Com2**) CT complexes can be visually observed for the former, but only spectroscopically studied for the later (Fig. 2). Firstly, stock solutions of 2 mM NDI-EA, DAN1 and DAN2 were prepared in DMSO, and then combined at 1:1 *v/v* ratio to give Complex 1 (**Com1)** and Complex 2 (**Com2)**, respectively. Typically, mixing pale yellow NDI-EA with colourless DAN1 in a 1:1 ratio gives a dark purple-reddish colour. Importantly, the mixing 1:1 NDI-EA with colourless DAN2 did not show any visual colour changes.

The UV−vis absorption spectrum of NDI-EA (aqueous 1 × 10^−5^ M, from 2 mM DMSO stock) showed a typical vibronically saturated spectra with two well resolved sharp absorption peaks at 387 and 364 nm along with a shoulder at 347 nm, which is characteristic of the S0 to S1 transition (Fig. 2A). It can be clearly seen that incremental addition of DAN1 (0–4 equiv.) resulted in a reduction in peak intensity along with an appearance of three new blue-shifted absorption peaks at 324, 310, 293 nm, respectively. This blue shift results from the hypochromic effect i.e. H-type aggregates formed in polar solvents due to increasing DAN concentration. The corresponding change in the shape of the H-aggregates also results from protonation of the side amino-group of NDI-EA in the presence of DAN-1. Furthermore, the appearance of a broad band over the range 400–900 nm for **Com1** is associated with the CT complexation process as shown in Figure S1 (ESI). However, such significant changes were not observed when DAN2 was gradually added to the NDI-EA solution at the same ratios (Fig. 2B). The reduction of peak intensity is not only due to formation of the CT complex of **Com1**, but also suggests the formation of larger supramolecular aggregates via side-by-side π-stacks of NDI (acceptor) chromophores and DAN1 (donor). A similar effect was observed for the case of H-aggregates in anomalous assemblies^28^. The binding constants (*K*_a_) of CT complexes of **Com1** and **Com2** were calculated on the basis of the Benesi–Hildebrand plot and found to be 1.45 × 10^4^ M^-1^ and 0.094 × 10^4^ M^-1^, respectively as shown in Figure S2 (ESI).

### Photophysical studies

The photophysical behaviour was firstly investigated by recording the fluorescence measurements in water (Figure S3 in ESI). Upon exciting the NDI-EA with 390 nm light, the emission bands of the singlet states of NDI-EA were observed with a maximum at 431 nm. These emission bands exhibited blue shifts upon titration with DAN1 due to complex formation between NDI-EA and DAN1. Further increasing the DAN1 to NDI-EA ratio resulted in significant fluorescence quenching of the emission band at 413 nm, which can be assigned to fast electron transfer from the DAN1 to the singlet-excited state of NDI-EA. Similarly, the fluorescence showed an approximate 20 nm blue shift for the Com2 complex during DAN2 to NDI-EA titration. The emission band at 413 nm showed no significant quenching upon further increase of the DAN2 to NDI-EA ratio, which suggests no electron transfer between NDI-EA and DAN2 at this specific wavelength.

Femtosecond transient absorption spectroscopy was used to obtain further insight into the excited state interactions of the **Com1** complexe. To this end, the differential absorption spectrum of NDI-EA control was first examined by excitation at 390 nm (Figure S2). The transient absorption bands observed at 10 ps, with maxima at 481, 609, 696, and 720 nm are assigned to the formation of the singlet excited state of NDI-EA, which decayed to the ground state with a rate constant of 6.7 × 10^11^ s^–1^ (lifetime: 1.5 ps)^29^.

**Figure 3:**
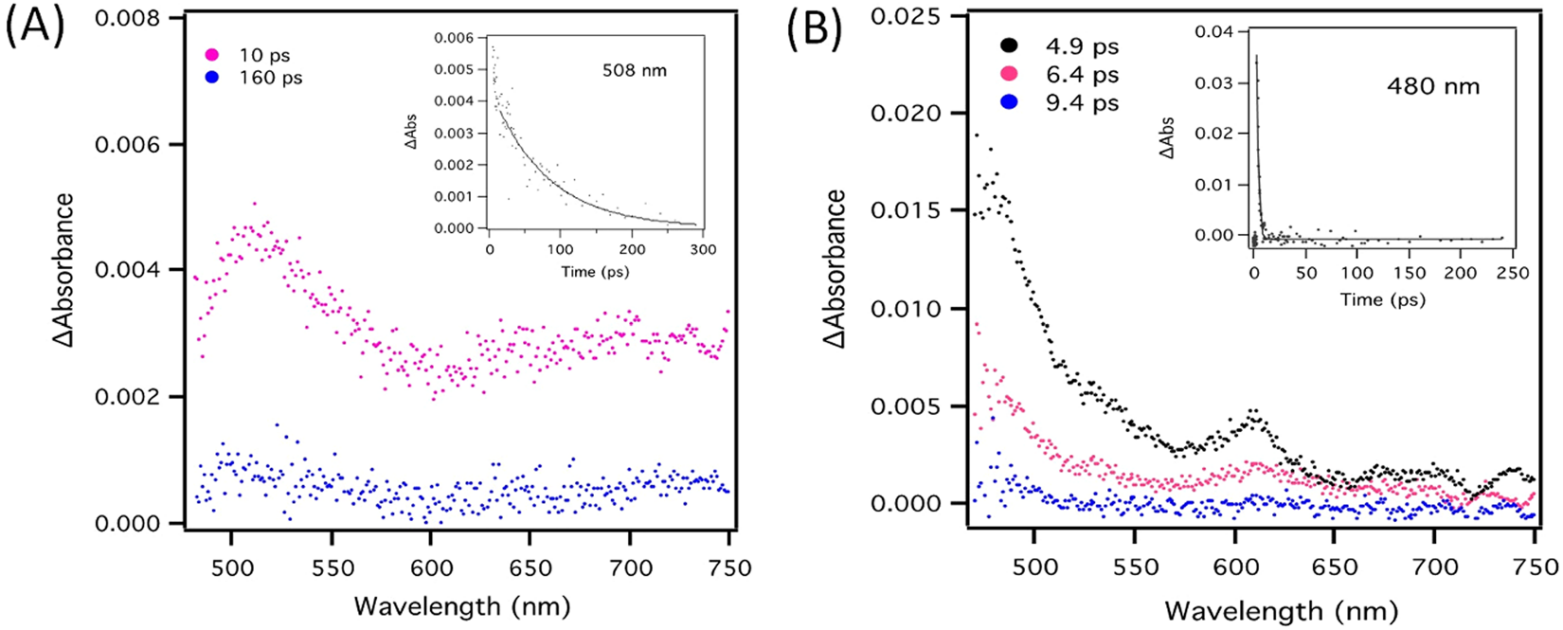
Photophysical studies: Differential absorption spectra obtained upon femtosecond laser photolysis of **Com1** (**A**); and **Com2** (**B**) in ultrapure water at the indicated time intervals.

The spectral features in the visible region, which are seen upon excitation of **Com1** in water (Fig. 3A) are quite different to that of the NDI-EA control (Figure S4). As seen in Fig. 3A, The transient spectra at 10 ps exhibited a strong absorption band at 508 nm, and the absorption extends over the whole spectra range employed (up to 800 nm). We attribute this to the NDI radical anion, which is well described in literature from previous electrochemical and photochemical studies^30,31^. The appearance of the NDI radical anion would imply that the singlet excited state decays via an electron-transfer (ET) process, in which DAN 1 acts as an electron donor and NDI-EA acts an electron acceptor. From the rise and decay of the NDI-EA radical anion at 508 nm, the rate constants of charge-separation and charge-recombination processes were found to 7.7 × 10^11^ and 1.64 × 10^10^ s^−1^, respectively. Based on the rate constant of charge recombination, the lifetime of the charge-separated state of **Com1** complex was determined to be 61 ps. When turning to **Com2** complex (Fig. 3B), it is interesting to note that the recorded femtosecond transient absorption spectra showed similar features to NDI-EA control, but is quite different to the **Com1** complex. As seen in Fig. 3, the spectra at different time scales showed only the formation of the singlet excited state of NDI-EA with maxima at 481, 611, 700 and 763 nm, with a rate constant of 6.7 × 10^11^ s^−1^, which is comparable to that observed of the NDI-EA control. This finding suggests no evidence of photoinduced electron transfer from DAN2 to NDI-EA in the complex.

### Electrochemistry

Electrochemical measurements of the complexes, and the individual molecules were undertaken in solution and in the solid state on an evaporated film, to track the self-assembly of the charge-transfer complex through changes in the redox activity of the precursors and assembled complexes. Electrochemical studies in solution revealed NDI-EA to be redox active, as expected, with the first reduction peak at approximately −0.29 V vs. Ag/AgCl. When DAN1 and NDI-EA were added in equal amounts a purple suspension formed, and the diffusion limited wave for the reduction of dissolved NDI-EA was suppressed, due to the lack of free NDIED in solution. Drop casting of this purple suspension onto a glassy carbon electrode, and sub-sequent solid state voltammetry in aqueous NaClO_4_ revealed significant differences in the profile compared to solid state voltammetry of drop cast NDI-EA. Figure S5 shows the overlay of the solid state voltammetry of the purple complex and the NDI-EA straying material. DAN1 is not redox active in this potential range. It is seen that the voltammogram is significantly different, indicating that the electronic properties of the NDI-EA has been significantly altered, possible through a conformation that includes charge transfer. The optical properties of this material more precisely describe the charge-transfer characteristics, however, the reduction wave for the NDI-EA in the CT complex in the solid state is significantly shifted.

### Theoretical Calculation

This spectroscopic disparity between **Com1** and **Com2** drove us to conduct a theoretical modelling study of these two complexes. Individual molecules (NDI-EA, DAN1 and DAN2) were energy optimized in the gas phase using the Gaussian 16 suite of programs at the TD-DFT b3lyp/6–311 + g(d,p) level of theory. The molecular modelling of the unsubstituted aromatic NDI-EA and DAN cores, in general, gives a quadrupole moment with partial negative charge off the molecular plane where the electron density of the π conjugated system is, and a partial positive charge in plane around the periphery (see Fig. 1). This quadruple charge distribution is reversed when the aromatic system is substituted with electron-withdrawing groups, giving a partially positively charged centre and a negatively charged periphery. Here, the positively charged centre of NDI-EA is further increased by the ethylamine N-substitution groups, which also may be protonated. DAN1 and DAN2 are substituted with alkyl phosphonate functional groups which can be deprotonated to result in further concentration of the negative charge on the naphthalene aromatic core. These arrangements potentially can result in a stronger electrostatic π-π interaction, or more accurately, donor – acceptor association, which is carried out via the aromatic π cloud. Nevertheless, the ionic interactions between the protonated amine and deprotonated phosphonate is much stronger than the partially charged aromatic systems. This can strongly affect the conformation of the complex, overriding the effects that the π-π interaction can induce.

**Figure 4:**
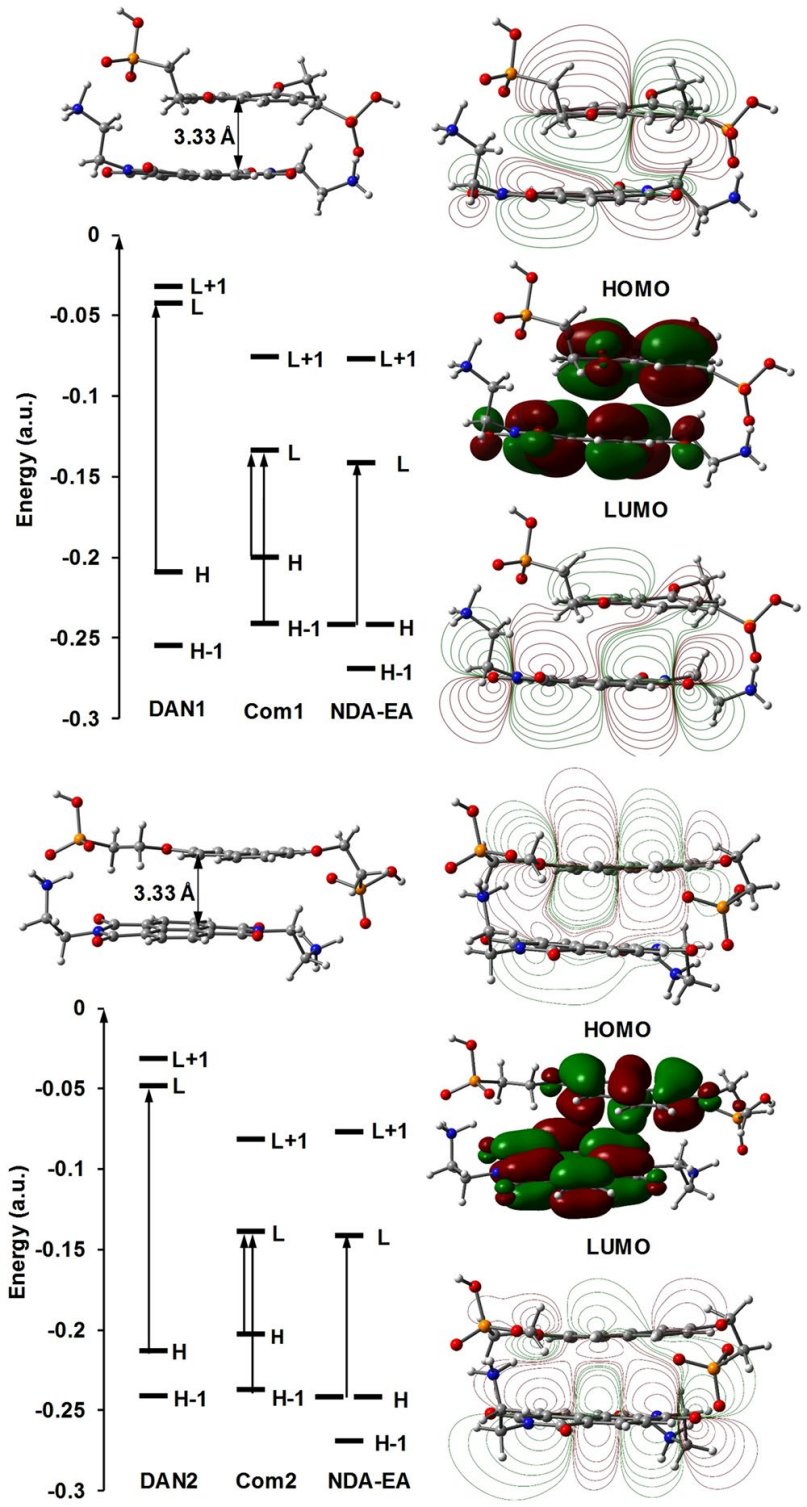
Theoretical Calculation: HOMO and LUMO electronic states of DAN1, DAN2, NDI-EA and **Com1** and **Com2** CT complexes as calculated using TD-DFT b3lyp/6-311 + g(d,p) level of theory.

To model the **Com1** and **Com2** complexes, we initially geometry optimized the models using the semi-empirical method of PM6 in the gas phase. This minimization gave the **Com1** complex structure, where the aromatic cores of DAN1 and NDI-EA stacked parallel with off centre shift and a distance of 3.33 Å between the molecular planes (Fig. 4). We then applied DFT at the meta-GGA hybrid M06-2X level on this geometry. The M06-2X function has been a method of choice for the study of aromatic stacked systems in which mid-range interactions are important^31^. Fig. 4 shows the HOMO, which is concentrated on the DAN1 aromatic core, and the LUMO, which is concentrated on the NDI-EA aromatic core in the donor – acceptor **Com1** complex. The cross section of the HOMO and LUMO of the complex perpendicular to the molecular planes and through the overlapping region of the NDI-EA and DAN1 in the complex shows extensive π cloud mixing between the donor and acceptor parts. The total electron density distribution in the complex clearly shows the complementarity of the geometry in the donor acceptor structure and electron density, where the contour cross-section of electron density shows an overlap between the NDI-EA and DAN1 π clouds (ESI Figure S6).

The geometry optimization of **Com1** and **Com2** at the ground state gave molecular structures reflecting the steric hindrances and the strength of the interaction forces present in the co-assembly of the acceptor (NDI) and the donor (DAN) molecules, but resulted in equal total energy as calculated using DFT at the meta-GGA hybrid M06-2X//6-31 + G(d,p) level of theory, which is a method of choice for the study of aromatic stacked systems^32^. Equal but slightly different total energy was obtained when calculations were conducted using TD-DFT b3lyp/6-311 + g(d,p) and TD-DFT b3lyp/6-311 + g(d,p) scrf = (solvent = water) levels of theory.

The 2,7 positions of the alkoxy substitutions in DAN2 give a longer distance between the phosphonate groups (Figures S7 and S8). Therefore, in order to maximize the stability produced by electrostatic interactions between the oppositely charged amine and phosphonate in **Com2**, the aromatic cores twist in and out of plane when compared to the parallel aromatic planes in **Com1**. The MO symmetry on the aromatic cores of the acceptor and donor molecules, and the difference between **Com1** and **Com2** conformations causes the hybridisation of donor and acceptor moieties MOs to result in different hybrid orbital symmetries. The symmetry of electronic states on the donor and acceptor moieties in the complexes determines the oscillation strengths of the allowed transitions, including the charge-transfer excitations.

### Electron Microscopy

**Figure 5:**
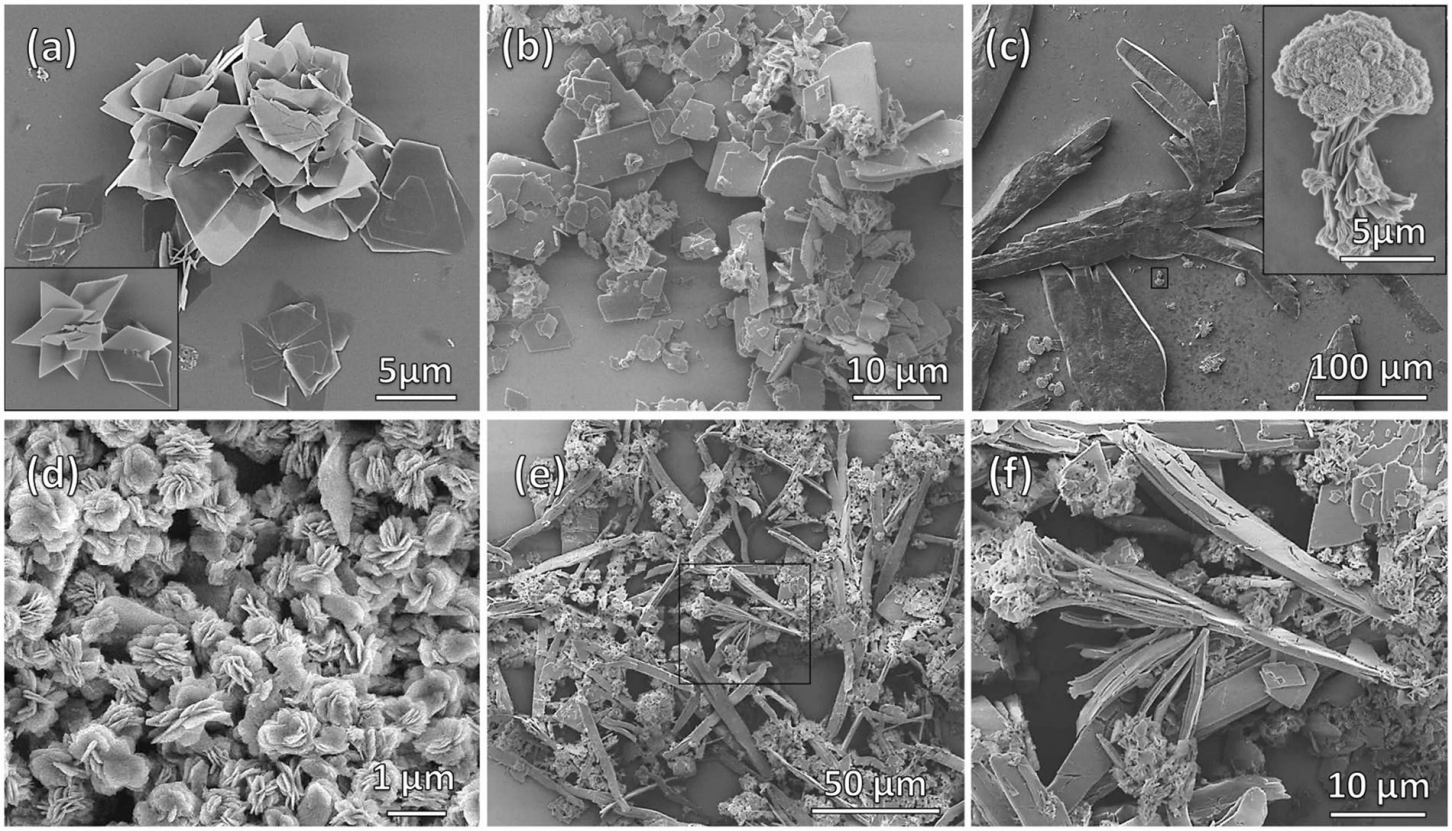
Electron Microscopy: SEM micrographs of the pure (**a**) NDI-EA, (**b**) DAN1, and (**c**) DAN2, and 1:1 mixtures of (**d**) **Com1** and (**e**,**f**) **Com2** deposited from MeOH-CH_2_Cl_2_ solutions on silicon wafer substrate.

Scanning Electron Microscopy (SEM) images show that all three compounds, NDI-EA, DAN1 and DAN2 are highly crystalline when deposited from MeOH/CH_2_Cl_2_ (1:1, *v/v*) solutions on their own. Very thin rhombic flakes were observed for DAN1, while thicker crystals with a more irregular structure were observed for DAN2, on average both about 5 μm in size (Fig. 5a,b). For NDI-EA, larger crystals are observed to grow, with structures that are hundreds of microns in length and tens of microns in width (see Fig. 5c), while smaller cauliflower like fractal structures were also observed for NDI-EA as shown in the inset in Fig. 5c.

**Figure 6:**
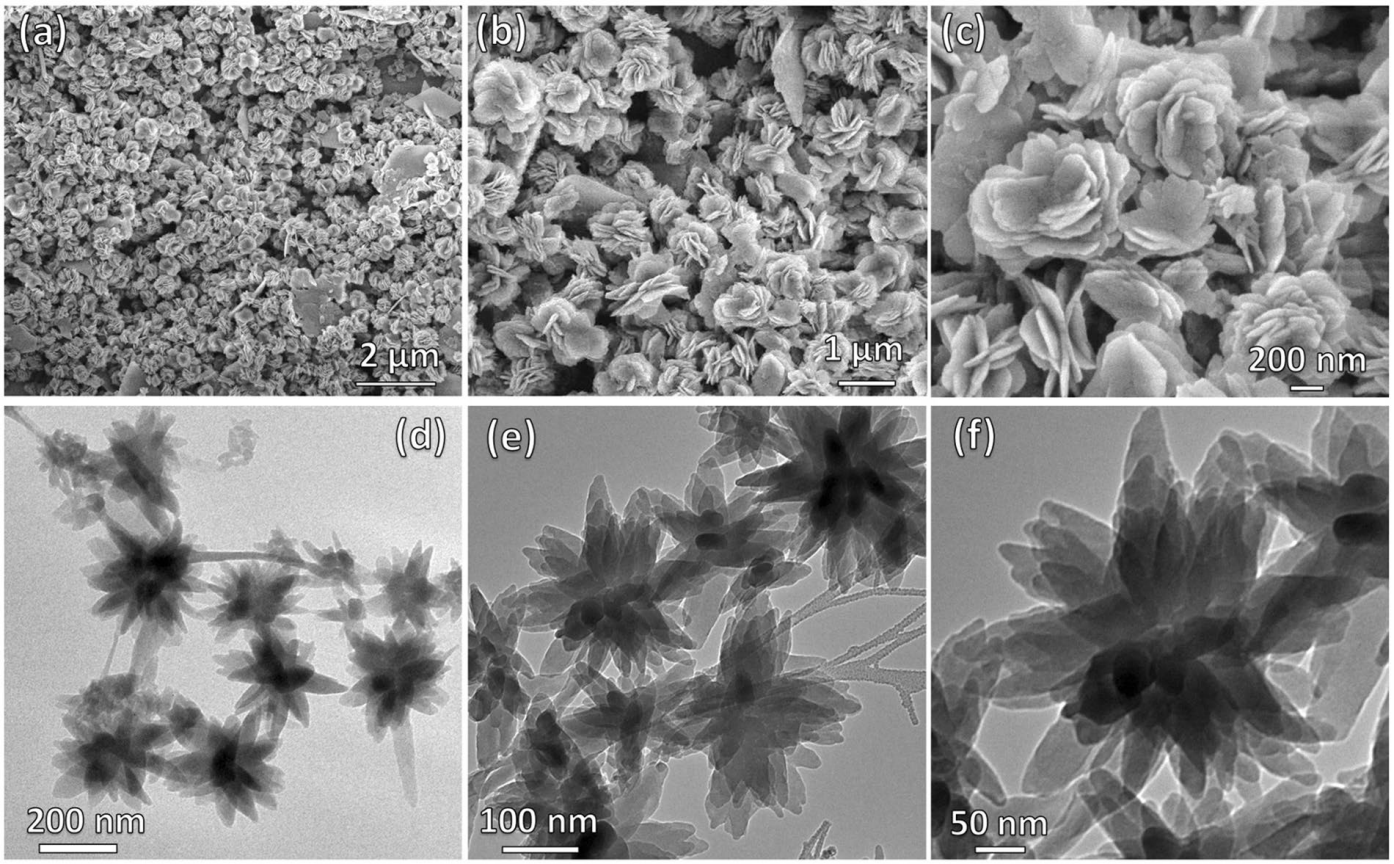
Electron Microscopy: Flower-like self-assembly at various magnifications: SEM (**a**–**c**) and TEM (**d**–**f**) micrographs of the **Com1** (1:1, *v/v* at ca. 10^−5^ M).

Interestingly, when NDI-EA and DAN1 solutions in MeOH/CH_2_Cl_2_ (1:1) were mixed in a 1:1 molar ratio, they assembled to form quite a uniform flower-like microstructure upon solvent evaporation. These flower-like superstructures are about 1 μm in diameter, and are composed of many paddles grown around a central point with a small inward curve. The flakes forming paddles of the flower-like superstructure are approximately 200–300 nm in size, and tens of nanometers in thickness (Figs 5d and 6a–c). However, when NDI-EA and DAN2 were dissolved and deposited using the same procedure, they produce large layered crystalline structures of very different morphology. Figure 5e shows that the the molecular geometry of DAN2 and NDI-EA does not allow for an optimum space filling alternating mixed stacking arrangement, suggesting that a segregated stacking of alternating molecular layers of NDI-EA and DAN2 produce the crystalline structures observed. The flaky texture of the material also suggests an in plane weakness which causes the peeling off of these layered sheets due to the hindrance that exists in the donor acceptor interaction between molecules residing in adjacent sheets. This causes the **Com2** crystal structure to inherit features from both the NDI-EA and DAN2 crystal structures. Figure 5f shows rhombic flakes similar to the structures deposited from pure DAN2, and a larger crystal-line structure similar to those of pure NDI-EA. This confirms that co-assembly of NDI-EA and DAN1 due to favourable geometry of the two molecules gives an energetically favourable superstructure with a morphology that is different from the precursors. Transmission Electron Microscopy (TEM) images of a 1:1 *v/v* mixture of the NDI-EA and DAN1 produced crystalline flower-like fractal structures when deposited on a carbon grid from water solution (Fig. 6d–f)^33^.

### X-ray Powder Diffraction (XRD)

**Figure 7:**
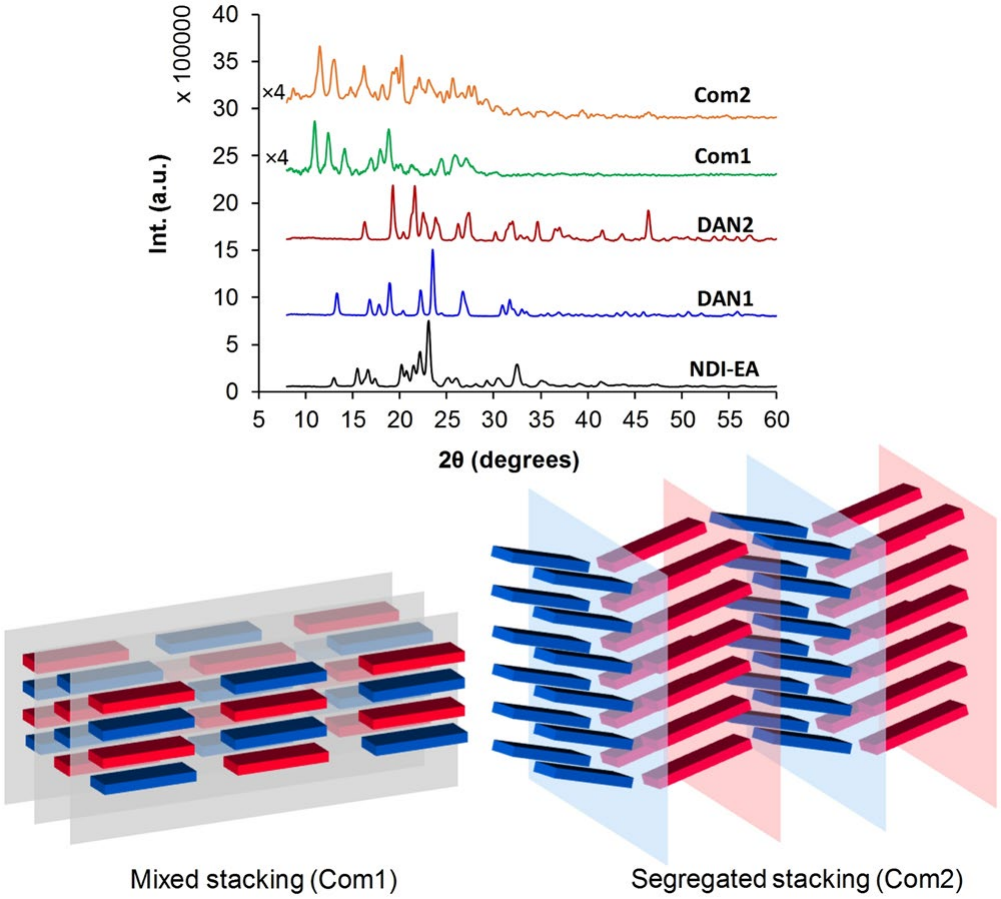
X-ray Powder Diffraction (XRD): X-ray powder diffraction of pure NDI-EA, DAN1, DAN2 and 1:1 ratios produced **Com1** and **Com2** which deposited from water solutions (The intensity of **Com1** and **Com2** patterns were multiplied by 4 for easy comparative visualization). The scheme shows the mixed and segregated stacking of **Com1** and **Com2** in the crystalline solid state (Red represent DAN and blue NDI cores).

Reczek *et al*. have studied the effect of saturated branched and unbranched peripheral substitution on the self-assembly and crystallization of NDI–DAN systems. They discovered that the crystallinity of these systems can be tuned *via* such substitutions^34^. In contrast, here we used substitutions with strong ionic interactions to affect the π-π stacking arrangement. X-ray powder diffraction data of **Com1** and **Com2** at 1:1 donor – acceptor ratios deposited from water solutions show much lower order in comparison to the precursors, which exhibit defined, strong reflections (Fig. 7). The experimental spectroscopic result shows no evidence of CT for **Com2** complex (Fig. 2), which suggest a segregated stacking of the NDI-EA and DAN2 in the solid state (Fig. 7).

The lack of charge transfer in **Com2** suggest no **DAN2** and **NDI-EA** cores stacking as schematically represented in Fig. 7, where in the less geometrically complementing molecules, although the molecular interactions are maximized to produce the most stable arrangement, a close packed alternating system cannot be produced due to the larger dimensions of **DAN2**. Instead the **Com2** arrangement results in alternating sheets of **DAN2** and **NDI-EA**, where the intramolecular interactions are maximized within sheets and between sheets. The molecular packing in **Com1** presents an alternating donor – acceptor order on one dimension while every molecule is surrounded by its counterpart from all directions in crystal motif. This is mainly due to the similar geometry and size of the two molecules and the complementing interacting functional groups and charge distribution giving higher order and smaller number of reflections in comparison to **Com2** in the XRD scattering pattern^35,36^.

FTIR spectroscopy of the **NDI-EA**, **DAN1** and **DAN2** and the complexes **Com1** and **Com2** reveals the various interactions between donor and the accepter in the complexes. Most importantly, the main interactions are in between the phosphonate and amine functional groups. The phosphonate group protonation state can be followed using the peaks at 937 and 1039 cm^−1^ for P-OH in its protonated form while the deprotonated phosphonate gives peaks at 914 and 1008 cm^−1^ wavenumbers. **DAN1** and **DAN2** both show a higher ratio of protonated phosphonate groups which lose a proton in the complex to the amine functional groups in the **NDI-EA** in the complex giving arise to the peaks at 914 and 1008 cm^−1^ in both complexes (See Figure S9). This proton transfer clearly can be observed in **Com2** where the geometry of the two molecules in the complex does not allow for the proton to position itself in a space that can be shared by the two functional groups. **Com2** shows a clear bending vibration for the NH_3_^+^ protonated amine at 1547 cm^−1^, while the amine peak of the pure **NDI-EA** at 1527 cm^−1^ has disappeared in **Com1**, and no sharp peak for the protonated amine can be observed. This broadening in the peak occurs when a strong hydrogen bonding between the protonated amine and deprotonated phosphonate results in effective sharing of the proton.

**Figure 8:**
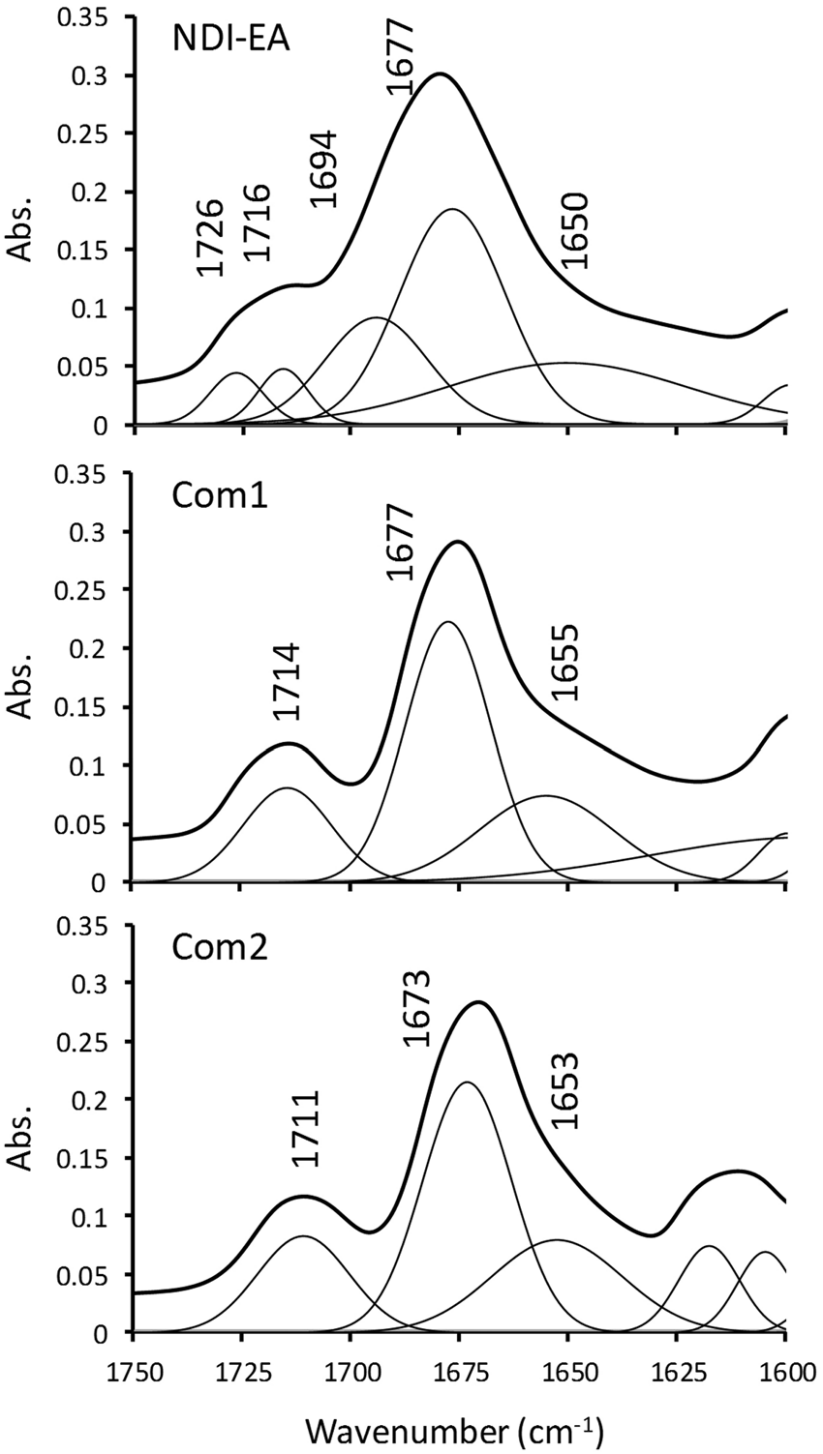
FTIR spectra deconvoluted: The deconvoluted FTIR spectra of NDI-EA, Com1 and Com2 in the C=O vibration range.

The self-assembly of the aromatic system is best reflected in the symmetric and asymmetric vibrations at 1677 and 1716 cm^−1^ of the C = O of the imide group of the **NDI-EA** (See Figure S9). The self-assembly of the pure **NDI-EA** results in shoulders in 1694 and 1726 cm^-1^ for the symmetric and asymmetric vibrations as can be seen in the deconvoluted peaks in Fig. 8. In peptide substituted NDIs these shoulders have been assigned to β-sheet structure in the assemblies, which can be assigened to similar arrangement in the **NDI-EA** self-assembly^37^. These shoulders have disappeared in both complexes resulting in sharper more defined peaks in the C = O region of vibrations. **Com1** complex showed no significant shift in the main peaks giving vibrations at the same wavenumbers of 1677 and 1716 cm^−1^ for the symmetric and asymmetric modes. The **Com2** complex has shown a 4 cm^−1^ shift to lower energies in both the symmetric and asymmetric vibrations. This FTIR shift indicative of stronger charge interaction between the **DAN** and **NDI** cores, which may be due to more effective charge separation in the amine and phosphonate moieties. The positive charge on the amine groups turns the **NDI** core to better acceptor and the negatively charge phosphonate turns the **DAN** core to a better donor in both complexes, this effect is even stronger in **Com2**, but the charge transfer only occurs of orbital symmetry on the donor and acceptor cores allow.

## Discussion

The appearance of peaks at lower angles in the diffraction patterns of the **Com1** and **Com2** indicates an increase in unit cell dimensions corresponding to the spacing induced by the alternating donor-acceptor arrangements in both **Com1** and **Com2**. The complex pattern in the **Com2** spectrum shows a low arrangement symmetry resulting from the twist in the π stacked alternating aromatic cores due to the hindrance applied by the strong electrostatic interaction of the periphery moieties, while the complementing geometry of the **Com1** resulted in much simpler spectrum^38^.

The two complexes were designed with the NDI aromatic core acceptor and two isomers of DAN with charge carrying moiety substitutions to stabilise the assembly via electrostatic interactions. The spectroscopy of the complexes shows that the CT is photoinduced, therefore we approached these CT complexes as a class II valence delocalised systems^39^, where the electron transfer occurs through space^40,41^. The local environment of the chemical structure is the main factor in inducing supramolecular assembly, especially via the secondary coordination sphere, where the intermolecular interactions determine the physical and chemical properties of a self-assembled material. One of the best examples of interdependency of chemical structure and functional behaviour are charge transfer complexes, where the assembly is functional only when the donor and acceptor molecules are positioned to form an orbital geometry that allows for electron transfer to occur. This is essential both in designed synthetic^3,4^ and biological^5–7^ systems. Solvation and solvophobic effects play a significant role in the aggregation process and the binding strength between the electron-rich donor and electron-poor acceptor molecules. Other properties of the solvent such as its electron donating capacity also have an important role in the electron transfer process^8^.

The polarization of the aromatic systems lays the basis for the energetic interaction between the two π systems. Face centred interactions between NDI and DAN can occur because of the complementary geometry of the quadruple polarization in the NDI, and the reversal quadruple polarization in the DAN aromatic system^11,13^. These alternating electron-deficient and electron-rich aromatic molecules can stack in an arrangement that results in the through space mixing of adjacent π orbitals, providing the basis for charge transfer between the two molecules. This charge transfer results in an absorbance band that can be assigned to excitation of an electron in the HOMO π orbital of the electron-rich molecule to the LUMO π orbital of the electron-acceptor moiety of the complex. This transition is due to the smaller energy gap of the donor’s HOMO and acceptor’s LUMO in the co-assembled complex in comparison to the HOMO – LUMO gaps in either species in isolation.

To the best of our knowledge, the CT complexation, along with the formation of flower-like structures in both aqueous and organic medium described herein is the first example of such assembly being observed in a supramolecular system containing two organic components i.e. acceptor (NDI appended diamine NDI-EA) and donor (phosphonic acid-substituted DAN1). In this case, diamine NDI (**1**) possesses two important properties resulting in the formation of a controlled CT complex and flower-like nanostructures: (i) the aromatic core of the NDI, which is ideal to maximise dispersive interactions (π–π stacking and van der Waals interactions) between the perfectly matching donor (DAN)-acceptor (NDI-EA) in the charge transfer complex and (ii) the amino group of NDI interacts with the phosphonic acid group of DAN through strong hydrogen-bonding (H-bonding) and electrostatic interactions. Formation of the CT complex of NDI-EA with DAN1 prevents crystallization and favours the directional growth of a flower-like superstructure in a 3D fashion, which was further evidenced by using the mismatching DAN2 (which does not produce a CT complex or any supramolecular assemblies).

Furthermore, this study also reveals an important factor in CT-complex formation, as DAN2 bearing phosphonate groups at 2,7- positions mismatches for electrostatic interactions with the amino groups of the NDI acceptor, preventing formation of a CT complex. Therefore, this study clearly indicates that not only aromatic interaction but also electrostatic interactions are important.

In conclusion, we exemplify the effect of conformation of the molecules involved in organic complexes on the charge transfer transition permission. XRD spectra show that despite similar molecular distances in the crystal structures of **Com1** and **Com2**, the relatively longer molecule of **DAN2** is forced to self-assemble in segregated form with angle twist in relation with **NDI-EA** to achieve the optimum packing density and maximum stabilizing interactions. This segregation and twist renders the charge transfer between the donor (**DAN2**) and acceptor (**NDI-EA**) forbidden, as observed in UV-vis spectra and the time resolves spectroscopy studies. Therefore despite theoretical calculations showing similar bandgaps for both **Com1** and **Com2**, we observe a higher oscillation strength for the charge transfer transition Com1 (*f* = 0.0056) in comparison to **Com2** (*f* = 0.0028) in vacuo even in the model where the aromatic cores are not segregated. The charge transfer transition occurs in the H-1 → L after the protonation and deprotonation of the amine and phosphate moieties in **NDI-EA** and **DAN1** and **DAN2** molecules.

## Materials and Methods

### General Methods & Materials

Naphthalene-1,5-diol, naphthalene-1,6-diol, dibromoethane, triethyl phosphite, trimethylsilyl bromide, naphthalene dianhydride, ethylene diamine and Boc-anhydride were purchased from Sigma-Aldrich, Bengaluru, Karnataka, India. Acetonitrile and DMF were purchased from S. D. Fine Chemicals Limited (SDFCL). Dichloromethane and Methanol (AR grade) were purchased from Finar chemicals, India, and used without further purification. All reactions were maintained under a nitrogen atmosphere with dry solvents that were degassed for 10–15 min with nitrogen gas. Final compounds were purified using silica-gel column chromatography. ^1^H NMR and ^13^C NMR spectra were recorded on a Bruker Avance-400 MHz or 500 MHz spectrometer, or a Bruker Advance 100 MHz or 125 MHz spectrometer at 300 °K as stated. Chemical shifts (in ppm) were referenced to TMS (tetramethylsilane) as an internal standard. All measurements were performed in deuterated chloroform (CDCl_3_) or deuterated DMSO (as stated).

### Spectroscopic measurements

#### UV–Vis measurements

UV–Vis absorption spectra were recorded in a Cary-50, and UV–Vis spectrometer in 1 cm path length cuvette. A 0.2 mL aliquot of the stock solution of **Com1** and **Com2** (conc. = 10^−3^ M) from THF was transferred to various ratios ACN/THF in different volumetric flasks, and made up to 2 mL volume. The solutions were allowed to equilibrate for 2 h prior to the spectroscopic measurements.

#### Fluorescence Measurements

Fluorescence emission spectra were recorded on a Horiba Jobin Yvon FluoroMax®-4–Spectrofluorometer. Fluorescence measurements and quenching experiments were performed on a FluoroMax-4 equipped with an injector port and stirrer at 25 °C. All experiments were performed in a quartz cell with a 1 cm path length with 365 nm excitation wavelength.

#### Transmission Electron Microscopy (TEM) imagining

TEM samples were prepared by solvent evaporation on a holey carbon grid and micrographs were produced using a JEOL 1010 100 kV TEM.

#### Fourier Transform Infrared Spectroscopy (FTIR)

FTIR spectra were collected on a Perkin Elmer FT-IR 400 at ambient temperature. The instrument was continuously purged with CO_2_-free dry air.

#### SEM imagining

tock solutions of amino substituted naphthalene diimides **1** (c = 10^−4^ M) were prepared in Milli-Q water. A 0.2 mL aliquot of the stock solution of each one was transferred separately to four different volumetric flasks containing DAN1 (**2**) or DAN2 (**3**) (c = 10^–4^ M) with a 1:1 *v/v* ratio, respectively, and made up to a 2 mL volume with respective solvents. The solutions were allowed to equilibrate for 2 h prior to the Scanning Electron Microscopy (SEM) measurements. Silicon wafer substrates were used to deposited samples for SEM imaging. Substrates were cleaned with acetone, ethanol and then Milli Q water. SEM Samples were prepared by solvent evaporation on this silicon wafer and then sputter coated with gold for 10 s at 0.016 mA Ar plasma (SPI, West Chester, USA) for SEM imaging using a FEI Nova NanoSEM (Hillsboro, USA) operating at high vacuum which provided direct visualisation of the self-assembled aggregated structures.

#### TEM imagining

TEM samples were prepared by solvent evaporation on a holey carbon grid and micrographs were produced using a JEOL 1010 100 kV *Transmission Electron Microscope*.

## Electronic supplementary material

Supplementary Information
