# Supplementary material for: The sensitivity of donor – acceptor charge transfer to molecular geometry in DAN – NDI based supramolecular flower-like self-assemblies
Source: Sci Rep. 2017 Nov 28;7:16501. doi: 10.1038/s41598-017-15599-9 (PMC5705657; doi:10.1038/s41598-017-15599-9)
Supplement: Supplementary file 1 — Supplementary Information [file 41598_2017_15599_MOESM1_ESM.pdf]

Supplementary Information

The sensitivity of donor – acceptor charge transfer to molecular geometry in DAN – NDI based supramolecular flower-like self-assemblies

Mohammad Al Kobaisi,[1] Rajesh S. Bhosale,[2] Mohamed E. El-Khouly,[3,4] Duong Duc La,[1] Sachin D. Padghan,[2] Sidhanath V. Bhosale,[2] Lathe A. Jones,[1] Frank Antolasic,[1] Shunichi Fukuzumi[4,5] and Sheshanath V. Bhosale[1,]*

[1]School of Science, RMIT University, GPO Box 2476, Melbourne, VIC-3001, Australia. [2]Polymers and Functional Materials Division, CSIR-Indian Institute of Chemical Technology, Hyderabad 500007 Telangana, India. [3]Department of Chemistry, Faculty of Science, Kafrelsheikh University, Kafrelsheikh 33516, Egypt. [4]Department of Chemistry and Nano Science, Ewha Womans University, Seoul 03760, Korea. [5]Faculty of Science and Engineering, Meijo University, Nagoya, Aichi 468-8502, Japan.

Corresponding authors: Tel.:+61399252680; E-mail: sheshanath.bhosale@rmit.edu.au; bsheshanath@gmail.com

Photophysical studies

Fluorescence measurements were recorded using a FluoroMax specrtofluorometer (Horiba). Femtosecond laser flash photolysis was conducted using a Clark-MXR 2010 laser system and an optical detection system provided by Ultrafast Systems (Helios). The source for the pump and probe pulses were derived from the fundamental output of Clark laser system (775 nm, 1 mJ pulse-1 and fwhm = 150 fs) at a repetition rate of 1 kHz. A second harmonic generator introduced in the path of the laser beam provided 412 nm laser pulses for excitation. 95% of the fundamental output of the laser was used to generate the second harmonic, while 5% of the deflected output was used for white light generation. Prior to generating the probe continuum, the laser pulse was fed to a delay line that provided an experimental time window of 1.6 ns with a maximum step resolution of 7 fs. The pump beam was attenuated at 5 μJ pulse-1 with a spot size of 2 mm diameter at the sample cell where it was merged with the white probe pulse in a close angle (<10°). The probe beam, after passing through the 2 mm sample cell was focused on a 200 μm fibre optic cable, which was connected to a CCD spectrograph (Ocean Optics, S2000-UV-vis for visible region and Horiba, CP-140 for NIR region) for recording the time-resolved spectra (450-800 and 800-1400 nm). Typically, 5000 excitation pulses were averaged to obtain the transient spectrum at a set delay time. The kinetic traces at appropriate wavelengths were assembled from the time-resolved spectral data.

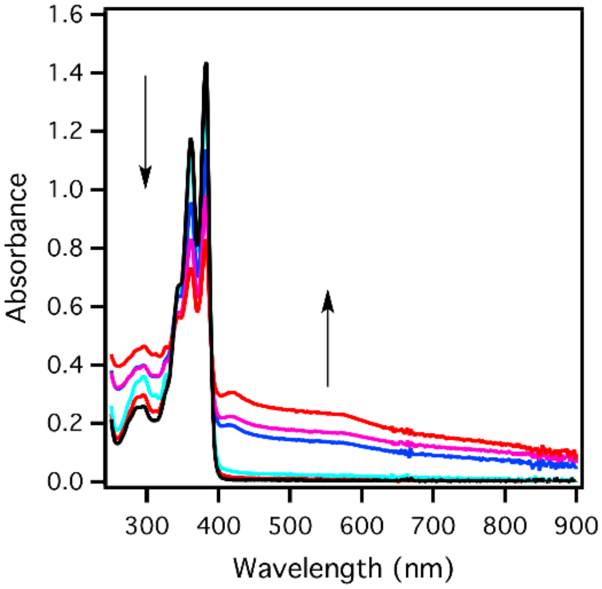

Figure S1. UV-Vis-NIR spectra of NDI-EA – DAN1 at various ratios i.e. 1:0.2 up to 1:1 molar ratio in aqueous solutions.

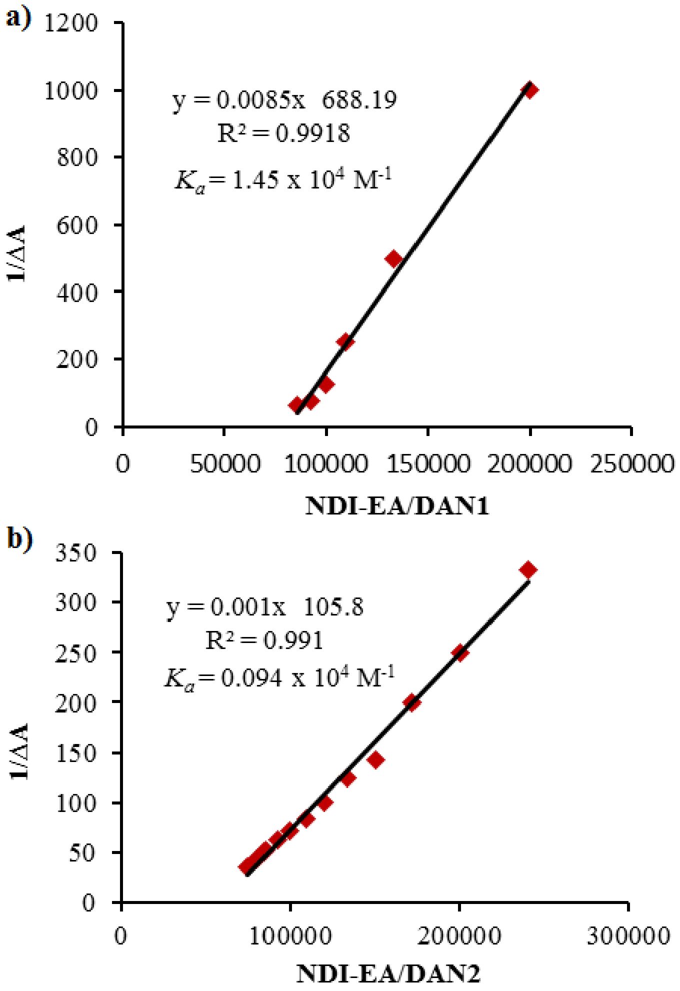

Figure S2. Benesi–Hildebrand linear plot for the CT complex of NDI-EA with: a) DAN1 and b) DAN2 from UV-vis absorption spectra in Figure 2 of the main manuscript.

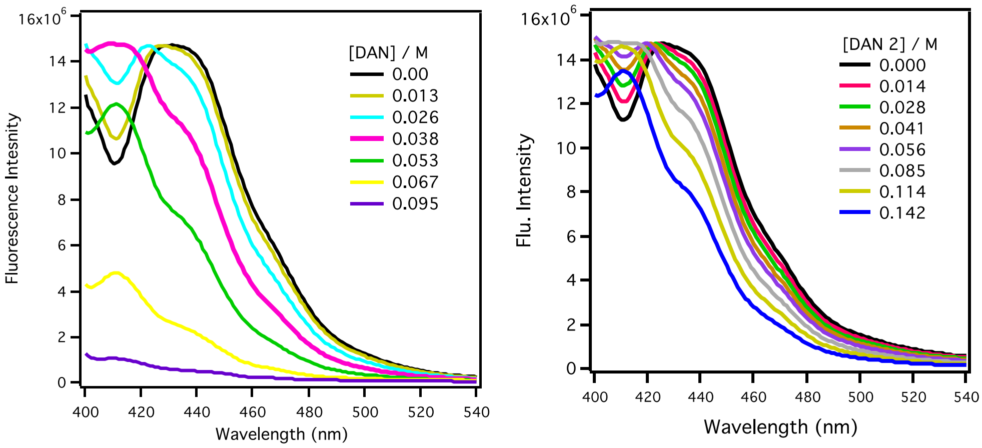

Figure S3. Fluorescence spectra of ***Com1*** *(NDI-EA – DAN1) (left figure) and* ***Com2*** *(NDI-EA – DAN2) (right figure) complexes in ultrapure water;* λ_{ex} *= 390 nm.*

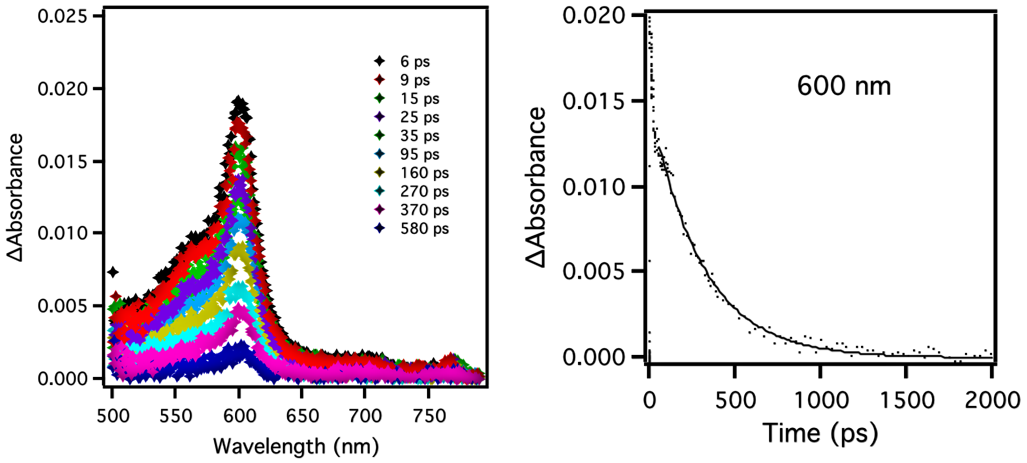

Figure S4. Differential absorption spectra obtained upon femtosecond laser photolysis of the NDI-EA control in ultrapure water at the indicated time intervals (Left figure). Decay profile of the NDI singlet state at 600 nm (Right figure).

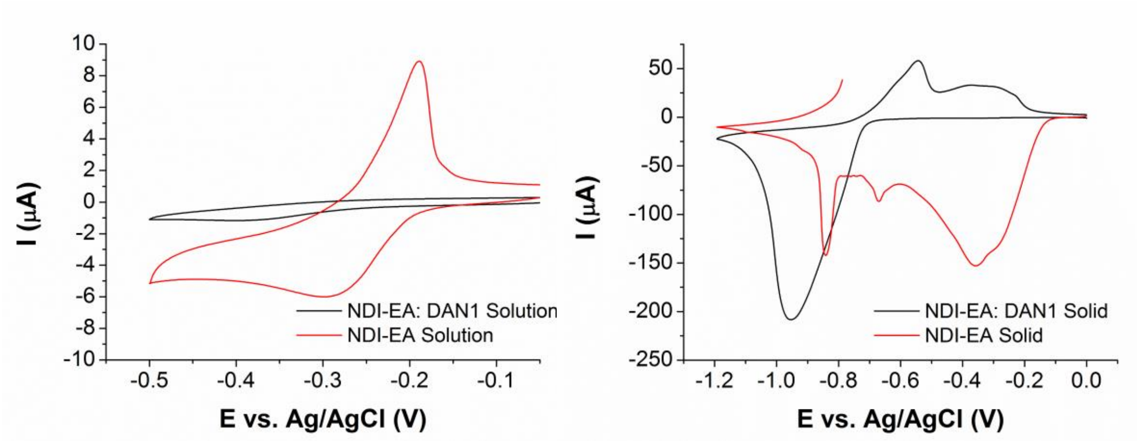

Figure S5. Voltamogram of NDI-EA in solution and 1:1 NDI-EA:DAN1 in solution (left) and dropcast NDI-EA and purple CT complex of NDI-EA:DAN1 on a glassy carbon electrode (right) in aqueous $NaClO_4$ *(right).*

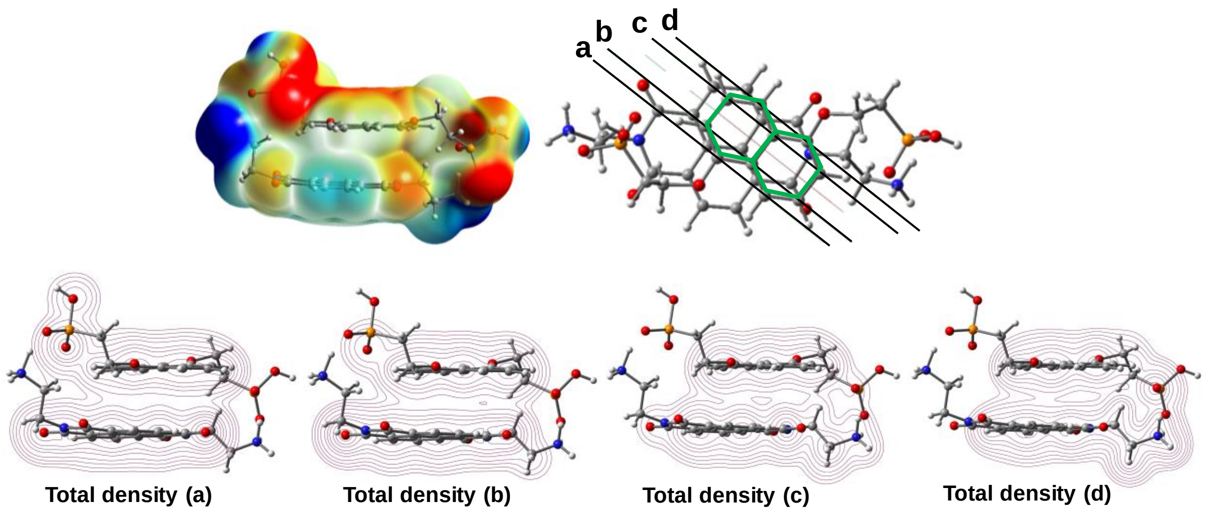

*Figure S6. Electron density shows an overlap between the NDI-EA and DAN1 π clouds (**Com1**).*

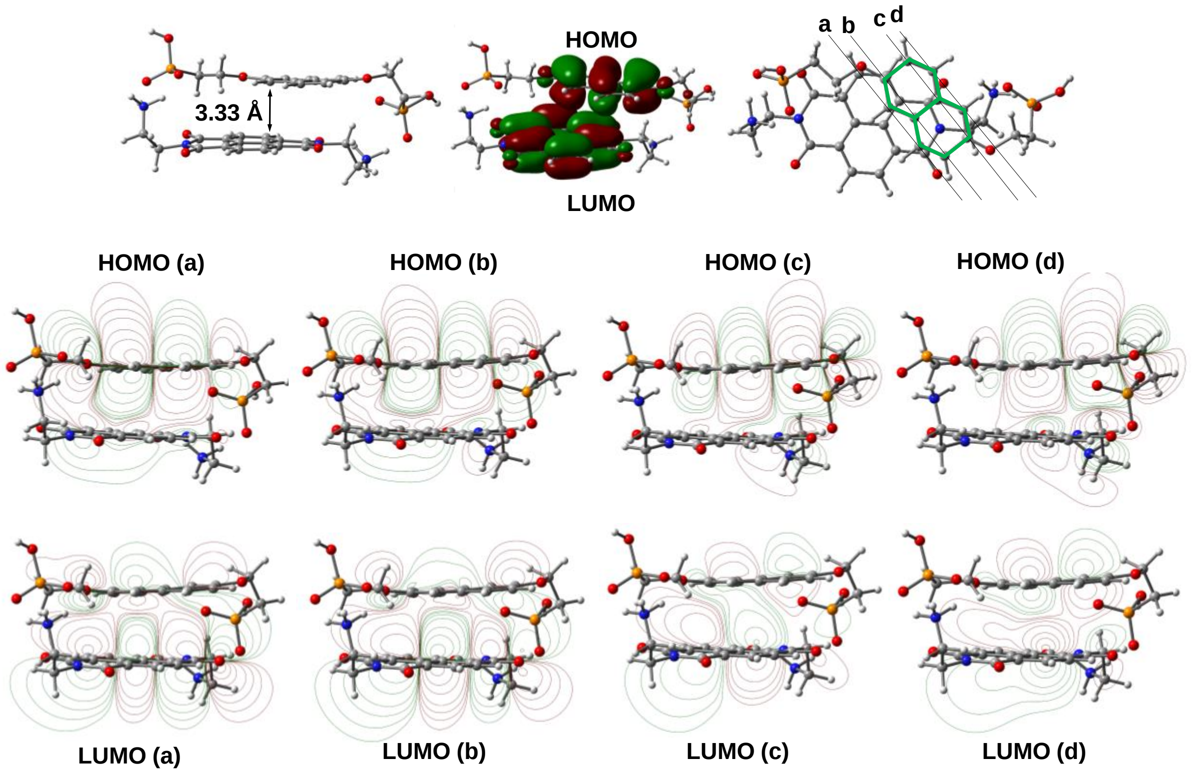

*Figure S7. The NDI-EA – DAN2 complex (**Com2**) HOMO and LUMO molecular orbitals cross-section contours showing the incomplete mixing of π clouds of the acceptor and donor and orbital symmetry.*

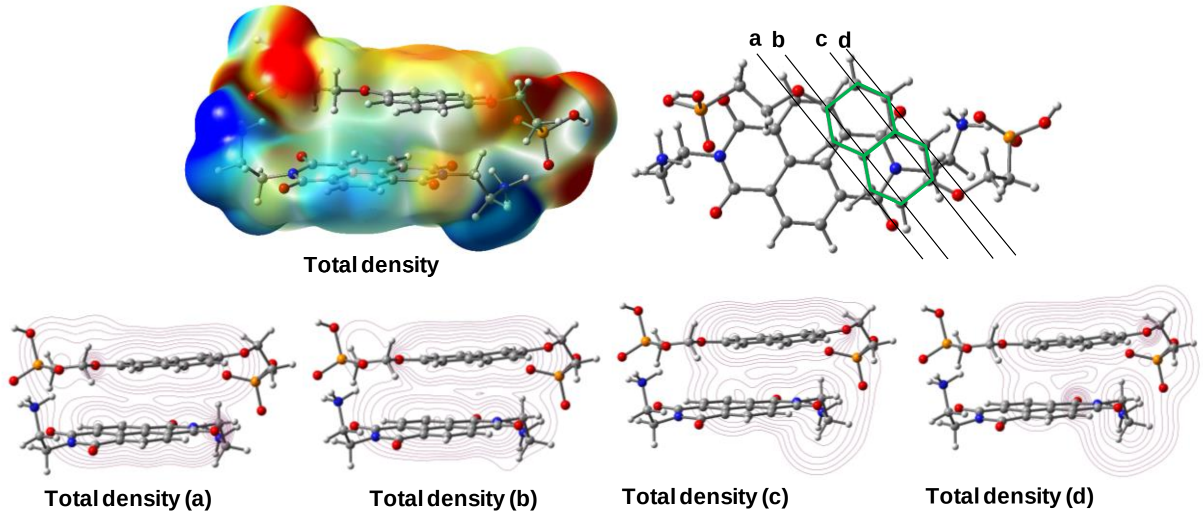

*Figure S8. The NDI-EA–DAN2 complex (**Com2**) total electron density distribution showing the effect of electrostatic interaction between the phosphonate and protonated amine groups on the molecular geometry resulting in fewer donors – acceptor π clouds overlap.*

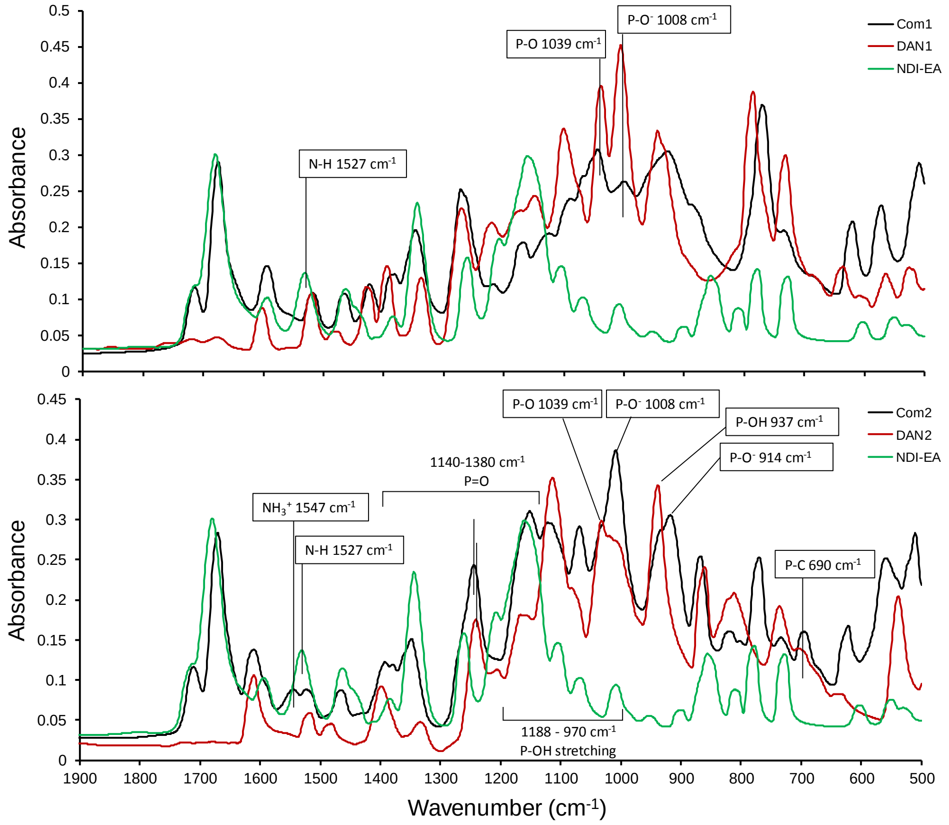

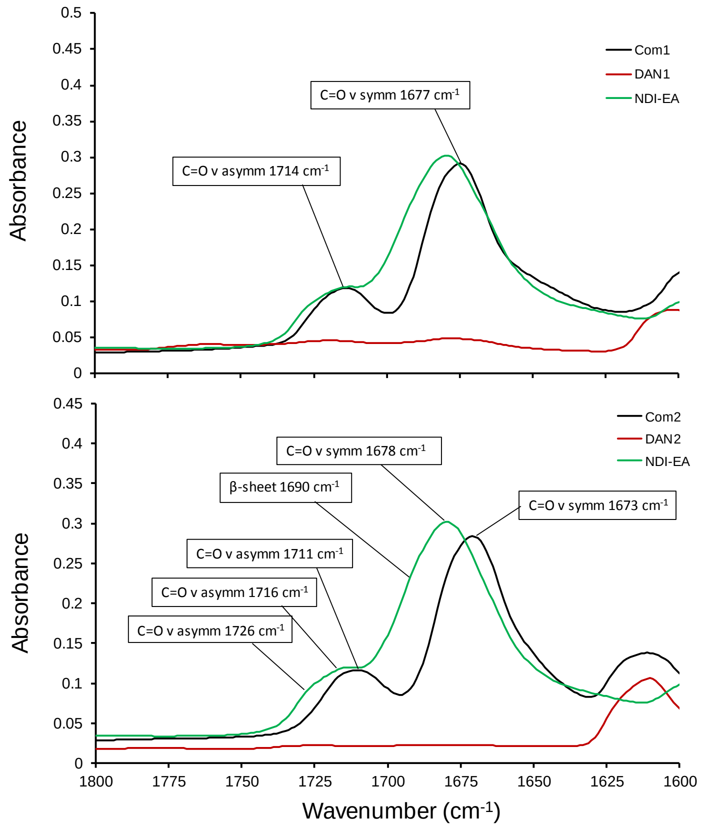

Figure S9. FTIR spectra of DAN1, DAN2, NDI-EA, Com1 and Com2, and an expended C=O vibrations range.

Experimental protocol for synthesis of target molecules

Synthesis of diamine naphthalene diimide derivative (NDI-EA).[S1]

NDI-EA (**1**) was synthesized in two steps, firstly by reacting mono Boc protected ethylenediamine with naphthalene anhydride followed by deprotection of the Boc group (Scheme S 1).

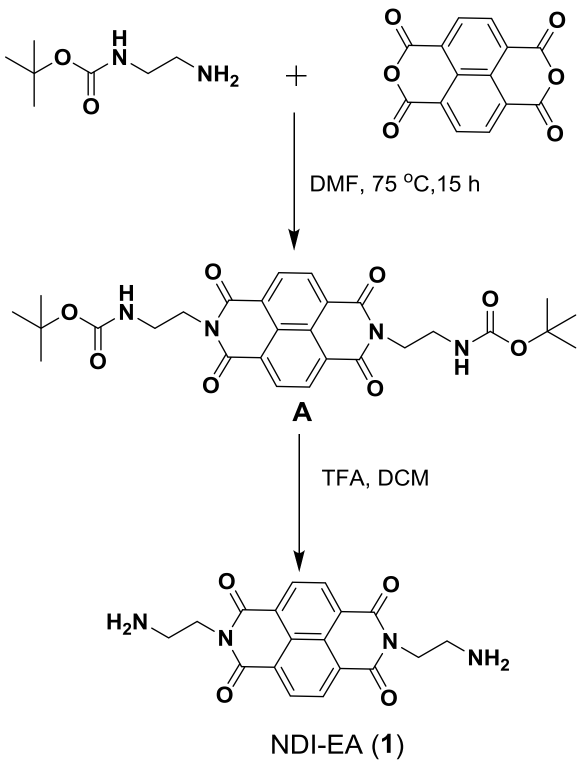

*Scheme S 1. Synthesis of NDI-EA (**1**)*

The mono Boc protected ethylenediamine (238.7 mg, 3.731 mmol) was added to 1,4,5,8-naphthalene tetracarboxylic dianhydride (500 mg, 1.865 mmol) in 15 mL of dry DMF (*N, N* dimethylformamide). The reaction mixture was stirred for 15 h at 75 ^{o}C. The progress of the reaction was monitored by TLC. The crude product was extracted using chloroform and concentrated under *vacuo*. The obtained crude product was further purified by column chromatography (1 % methanol in chloroform) producing a 75% yield. ^{1}H NMR (500 MHz, $CDCl_3$) δ: 1.23 (s, 18H), 3.53 (q, 4H), 4.36 (t, J=5.7 Hz, 4H), 5.30 (s, -NH, 2H), 8.76 (s, 4H).

Boc-removal:[S1] To a solution of **NDI 1** (100 mg) in dichloromethane maintained at 0 ^{o}C, TFA (trifluoroacetic acid; 3 mL) was added. The reaction mixture was stirred at room temperature for 3 h. The solvent was removed under reduced pressure using Rotary evaporation. The resulting brownish yellow solid was washed with toluene, then subsequently with chloroform and methanol. The obtained product was dried under *vacuo* to afford a white solid, **NDI-2** (70 mg). ^{1}H NMR (500 MHz, *dmso-d*^{6}) δ: 3.19 (t, J=5.4 Hz, 4H), 4.34 (t, J=5.7 Hz, 4H), 7.90 (s, **-NH$_2$**, 4H), 8.72 (s, 4H).

Synthesis of DAN1 (2) and DAN2 (3)

The synthesis of compound **2** ((naphthalene-1,5-diylbis(oxy))bis(ethane-2,1-diyl)) diphosphonic acid and compound **3** ((naphthalene-2,6-diylbis(oxy))bis(ethane-2,1-diyl)) diphosphonic acid were achieved by using a multistep synthetic strategy. As shown in Scheme 2, 1,5-dihydroxy naphthalene on etherification with 1,2-dibromoethane in dry acetonitrile yielded **4** 1,5-bis[2-bromoethoxy]naphthalene as white solid. Which was subsequently converted into compound **5** tetraethyl((naphthalene-1,5-diylbis(oxy))bis(ethane-2,1-diyl))bis (phosphonate) by treatment with triethyl phosphate at 110 °C. The target compound **2** was synthesized by the hydrolysis of phosphate functionality of compound **5** into phosphonic acid using trimethylsilyl bromide (TMSBr). The compound **3** was prepared in a manner analogous to that of **2**. The target compounds **2** and **3** are well characterised by using modern spectroscopic techniques such as ^{1}H NMR, ^{13}C NMR, ^{31}P NMR, mass and HRMS. The synthesis of naphthalene diimide (NDI-EA) **1** was synthesised following literature protocol.[S1]

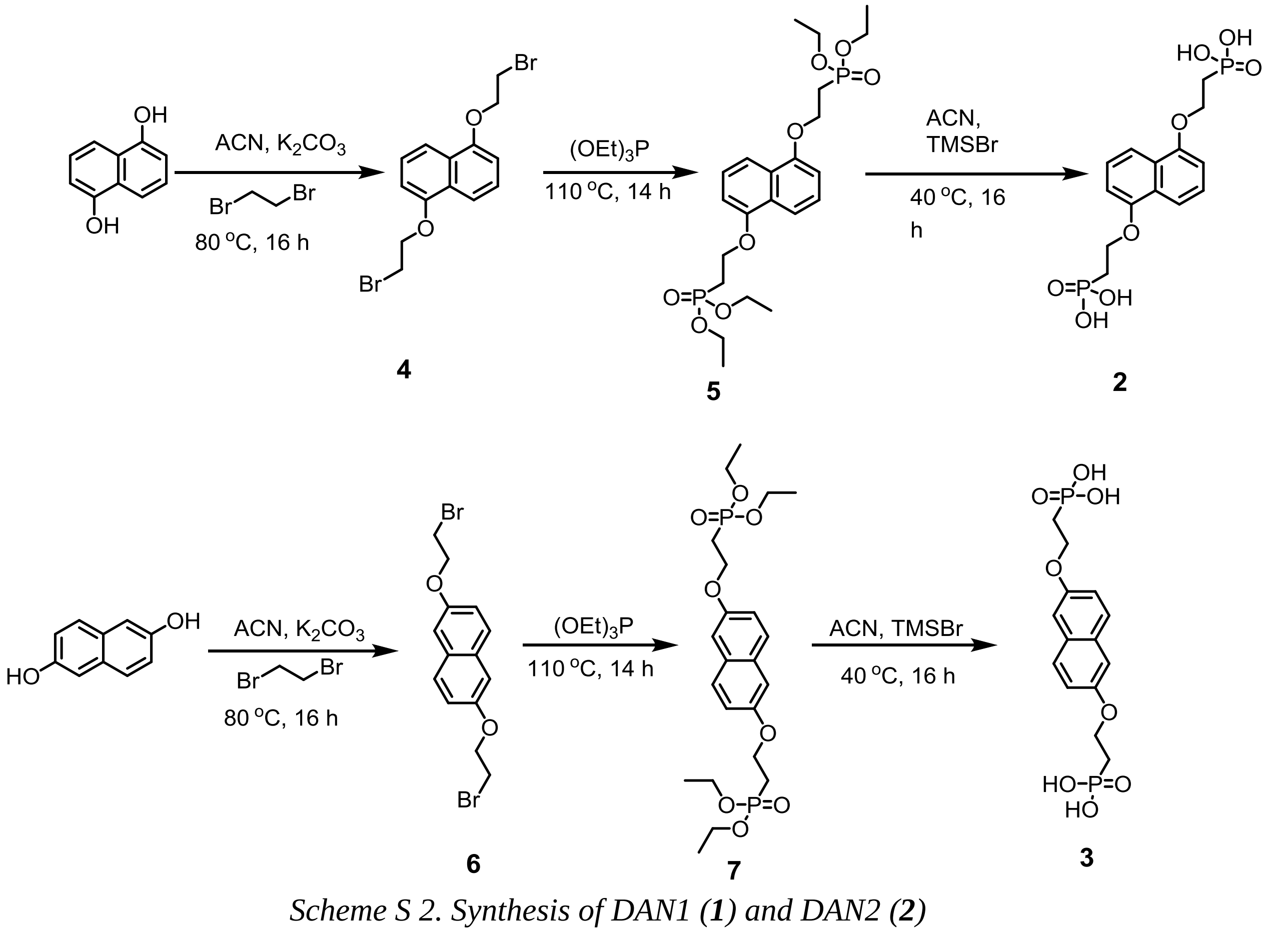

*Scheme S 2. Synthesis of DAN1 (**1**) and DAN2 (**2**)*

Synthesis of Compound 4

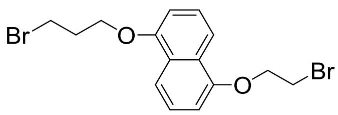

The solid 1, 5 dihydroxynapthalene (500 mg, 3.1 mmol) was dissolved in (15 ml) acetonitrile and K_2CO_3 (855 mg, 6.2 mmol) was added, the resulting suspension was stirred at 40 °C for 30 min. Then, 1, 2- dibromoethane (5 ml, 62.1 mmol) was added and the mixture was refluxed for 16 h. After cooling down and filtering of the solid, the solution was evaporated on a rotary evaporator. The residue was purified by silica gel column chromatography (EtOAc: Hexane 4:96) yielding **4** as a whitish solid (696 mg, 60%). Mp 164-166 °C. FT-IR (KBr, $^{-}$ v cm^{1}): 532, 577, 626, 770, 932, 968, 1028, 1090, 1264, 1378, 1411, 1510, 1592, 1713, 1824, 1917, 2855, 2930, 3057 and 3437; ^{1}H NMR ($CDCl_3$, 500 MHz) δ: 3.78 (t, 4H, J = 6.1 Hz), 4.45 (t, 4H, J = 6.2 Hz), 6.83 (d, 2H, J = 7.6 Hz), 7.37 (t, 2H, J = 7.9 Hz), 7.91 (d, 2H, J = 8.5 Hz); ^{13}C NMR (125 MHz, $CDCl_3$) δ: 29.2, 68.1, 106, 115.1, 125.2, 126.7, 153.6.

Synthesis of Compound 5

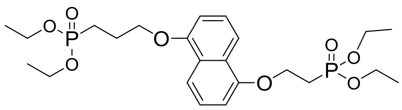

A mixture of compound **4** (200 mg, 0.53 mmol) and triethyl phosphite (2.5 ml, 2.1 mmol) was stirred at 110 °C for 14 h. The completion of the reaction was monitored by TLC. After completion of reaction the mixture was cooled to room temperature and the excess triethyl phosphite was removed under vacuum. The resulting residue was stirred in hexane for 1h to obtain crude solid. The crude solid was purified by silica gel column chromatography (CH_2Cl_2: MeOH 99:2) yielding **5** as a pale yellow solid (210 mg, 80%). Mp 115-116 °C. FT-IR (KBr, $^{-}$v cm^{1}): 511, 544, 707, 781, 954, 1020, 1237, 1265, 1387, 1421, 1512, 1593, 2904, 2982 and 3433; ^{1}H NMR ($CDCl_3$, 400MHz) δ: 1.34 (t, 12H, J = 7.1 Hz), 2.45 (m, 4H), 4.16 (m, 8H), 4.41 (m, 4H), 6.87 (d, 2H, J = 7.5Hz), 7.36 (t, 2H, J = 8.1 Hz), 7.87 (d, 2H, J = 8.4 Hz); ^{13}C NMR (100 MHz, $CDCl_3$) δ: 16.4, 27.3, 61.8, 62.3, 105.6, 114.6, 125.1, 126.6, 153.7; ^{31}P NMR (200 MHz, $CDCl_3$): 279.07; ESI-MS (m/z %): 489.7 (70) $[M + H]^{+}$; HRMS: calculated for $C_{22}H_{38}O_8NP_2$ = 506.20731 Found (ESI^{+}) $[M+ NH_4]^{+}$ = 506.20672.

Synthesis of Compound 2

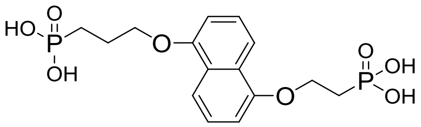

In an oven-dried 50 ml round bottom flask, compound **5** (100 mg, 0.20 mmol) was dissolved in dry acetonitrile (10 ml) and cooled to 0 °C. To this reaction mixture TMSBr (395 µl, 3.10 mmol) was added for 30 minutes at 0 °C and further stirred for 18 h at 40 °C. After completion of the reaction the reaction mixture was cooled to room temperature. The solvent was evaporated on a rotary evaporator and the resultant solid was stirred in 10 ml methanol for 2 h. The obtained precipitate was filtered and washed with 50 ml methanol to yielded compound **2** as a whitish solid (55 mg, 70%). Mp 258-261 °C. FT-IR (KBr, $^{-}$v cm^{1}): 465, 515, 630, 725, 781, 927, 942, 1001, 1031, 1090, 1141, 1213, 1260, 1328, 1386, 1417, 1512, 1594, 1706, 2327, 2887, 2929 and 3433; ^{1}H NMR (DMSO-*d*^{6}, 300 MHz) δ: 2.30 (m, 4H), 4.35 (m, 4H), 6.85 (d, 2H, *J* = 7.7 Hz), 7.33 (t, 2H, *J* = 7.9 Hz), 7.8 (d, 2H, *J* = 8.3 Hz); ^{13}C NMR (75 MHz, DMSO-*d*^{6}) δ: 29.5, 54.8, 107.1, 118.9, 128.2, 129.4, 154.5; ^{31}P NMR (200 MHz, DMSO-*d*^{6}):272.5; ESI-MS (*m/z* %): 377 (95) [M + H]$^{+}$, 399 (70) [M + Na]$^{+}$; HRMS: calculated for $C_{14}H_{19}O_8P_2$ = 377.05331 Found (ESI^{+}) [M + H]$^{+}$ = 377.05497, Calculated for $C_{14}H_{18}O_8NaP_2$= 399.03506 Found (ESI^{+}) [M + Na]$^{+}$ = 399.03691.

Synthesis of Compound 6

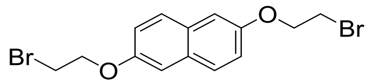

The solid 1, 6 dihydroxynapthalene (500 mg, 3.1 mmol) was dissolved in (15 ml) acetonitrile and K_2CO_3 (855 mg, 6.2 mmol) was added, and the resulting suspension was stirred at 40 °C for 30 min. Then, 1, 2-dibromoethane (5 ml, 62.1 mmol) was added and the mixture was refluxed for 16 h. After cooling down and filtering of the solid, the solvent was evaporated on a rotary evaporator. The residue was purified by silica gel column chromatography (EtOAc: Hexane 4:96) yielding **6** as a whitish solid (976 mg, 88%). Mp 168-171 °C. FT-IR (KBr, $^{-}$ v cm ^{1}): 475, 570, 685, 806, 855, 956, 1013, 1117, 1165, 1239, 1396, 1458, 1509, 1605, 1713, 1745, 1913, 2850, 2919 and 3436; ^{1}H NMR ($CDCl_3$, 500 MHz) δ: 3.70 (t, 4H, *J* = 6.2 Hz), 4.39 (t, 4H, *J* = 6.4 Hz), 7.10 (s, 2H), 7.16 (d, 2H, *J* = 8.8 Hz), 7.6 (d, 2H, *J* = 8.8 Hz); ^{13}C NMR (125 MHz, $CDCl_3$) δ: 29.1, 67.9, 107.5, 119.1, 128.4, 129.9 and 154.7.

Synthesis of Compound 7

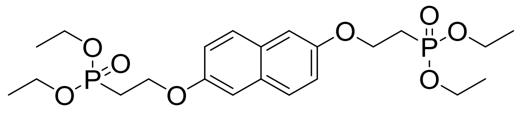

A mixture of compound **6** (200 mg, 0.53 mmol) and triethyl phosphite (2.5 ml, 2.1 mmol) was stirred at 110 ºC for 14 h. The completion of the reaction was monitored by TLC. After completion of reaction the mixture was cooled to room temperature and the excess triethyl phosphite was removed under vacuum. The resulting residue was stirred in hexane for 1h to obtain a crude solid. The crude solid was purified by silica gel column chromatography (CH_2Cl_2: MeOH 99:2) yielding **7** as a pale yellow solid (188 mg, 72%). Mp 114-116 °C. FT-IR (KBr, $^-$ v cm ^{1}): 479, 541, 626, 718, 776, 862, 962, 1075, 1117, 1166, 1224, 1243, 1393, 1509, 1604, 2922, 2977, 3432; ^{1}H NMR ($CDCl_3$, 500 MHz) δ: 1.36 (t, 12H, *J* =7.01 Hz), 2.37 (m, 4H), 4.16 (m, 8H), 4.33 (m, 4H), 7.11 (d, 4H, *J* = 5.7 Hz), 7.62 (d, 2H, *J* = 9.61 Hz); ^{13}C NMR (125 MHz, $CDCl_3$) δ: 16.4, 25.9, 27.3, 61.8, 107.3, 119, 128.2, 129.8 and 154.7; ^{31}P NMR (200 MHz, $CDCl_3$): 279.10; ESI-MS (*m/z* %): 489.7 (40) $[M+H]^{+,}$ 511.8 (100) $[M+Na]^+$; HRMS: calculated for $C_{22}H_{34}O_8NaP_2$ = 511.16211 Found (ESI^+) $[M + Na]^+$ = 511.16011.

Synthesis of Compound 3

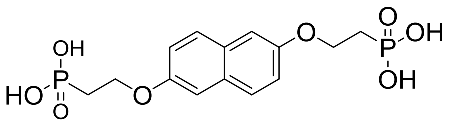

In an oven-dried 50 ml round bottom flask, compound **7** (100 mg, 0.20 mmol) was dissolved in dry acetonitrile (10 ml) and cooled to 0 °C. To this reaction mixture TMSBr (395 µl, 3.10 mmol) was added for 30 minute at 0 ºC and further stirred for 18 h at 40 ºC. After completion of the reaction the reaction mixture was cooled at room temperature. The solvent was evaporated on rotary evaporator and the resultant solid was stirred in 10 ml methanol for 2 h. The obtained precipitate was filtered and washed with 50 ml methanol to yielded compound **3** as a whitish solid (60 mg, 77%). Mp 288-291 °C. FT-IR (KBr, $^-$ v cm ^{1}): 533, 728, 809, 856, 934, 999, 1030, 1115, 1167, 1236, 1322, 1392, 1475, 1510, 1606, 1709, 2325, 2889, 2937, 3431; ^{1}H NMR ($CDCl_3$, 300 MHz) δ:2.12 (m, 4H), 4.23 (q, 4H, *J* = 7.74 Hz), 7.13 (d, 2H, *J* = 8.87 Hz), 7.25 (s, 2H), 7.76 (d, 2H, *J* = 8.87 Hz). ^{13}C NMR (75 MHz, DMSO-d^6) δ: 29.2, 62.9, 106.9, 118.9, 128.1, 129.5 and 154.5; ^{31}P NMR (200 MHz, DMSO-d^6):273.69; ESI-MS (*m/z* %): 377 (95) $[M+H]^+$; HRMS: calculated for $C_{14}H_{19}O_8P_2$ = 377.05343 Found (ESI^+) $[M+H]^+$ = 377.5497. Calculated for $C_{14} H_{18} O_8 Na P_2$= 399.03504 Found (ESI^+) $[M+Na]^+$ = 399.03691.

^{1}H NMR of Boc protected NDI-EA

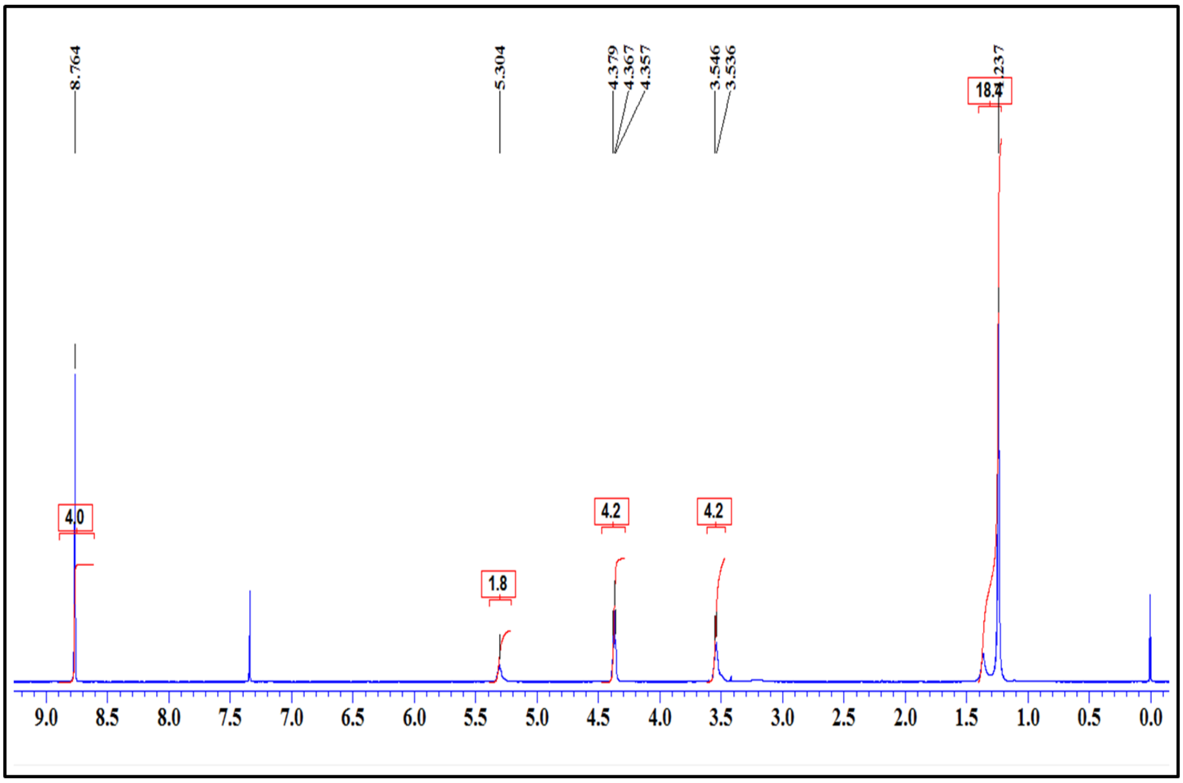

^{1}H NMR of NDI-EA

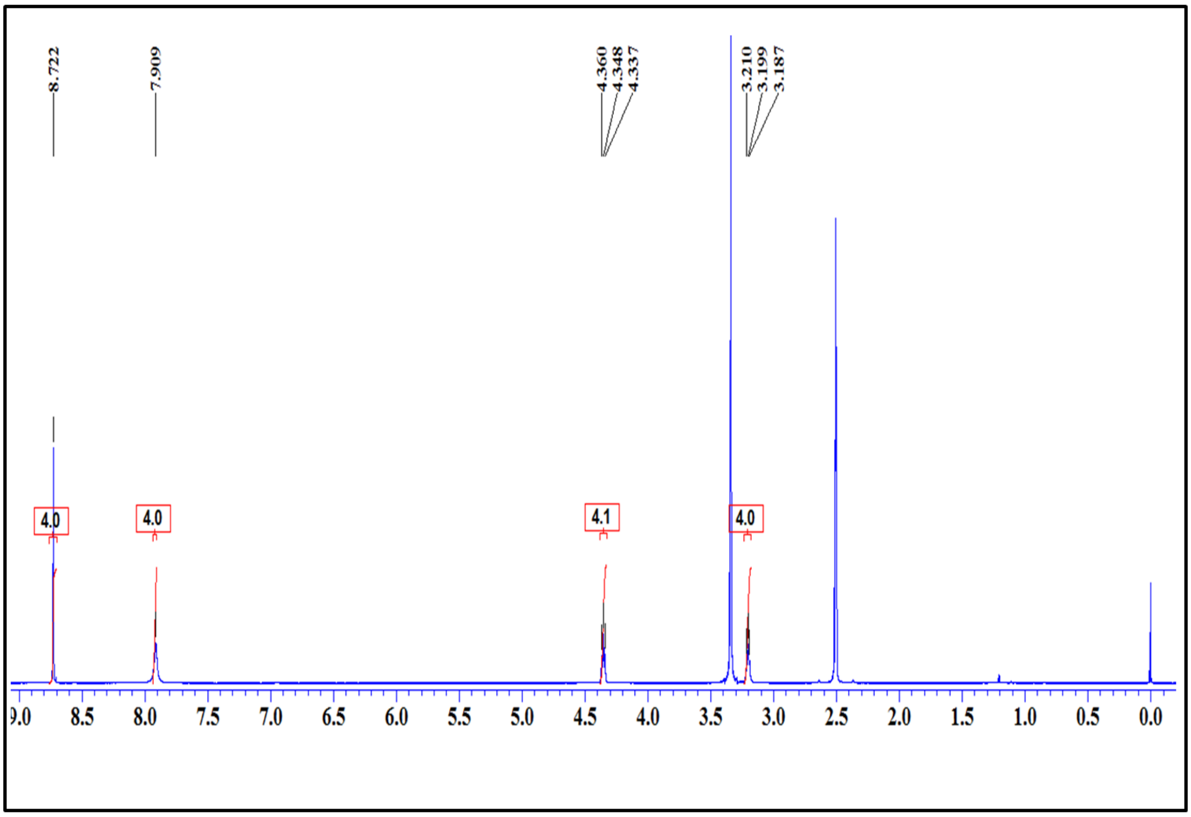

FT-IR of compound 4

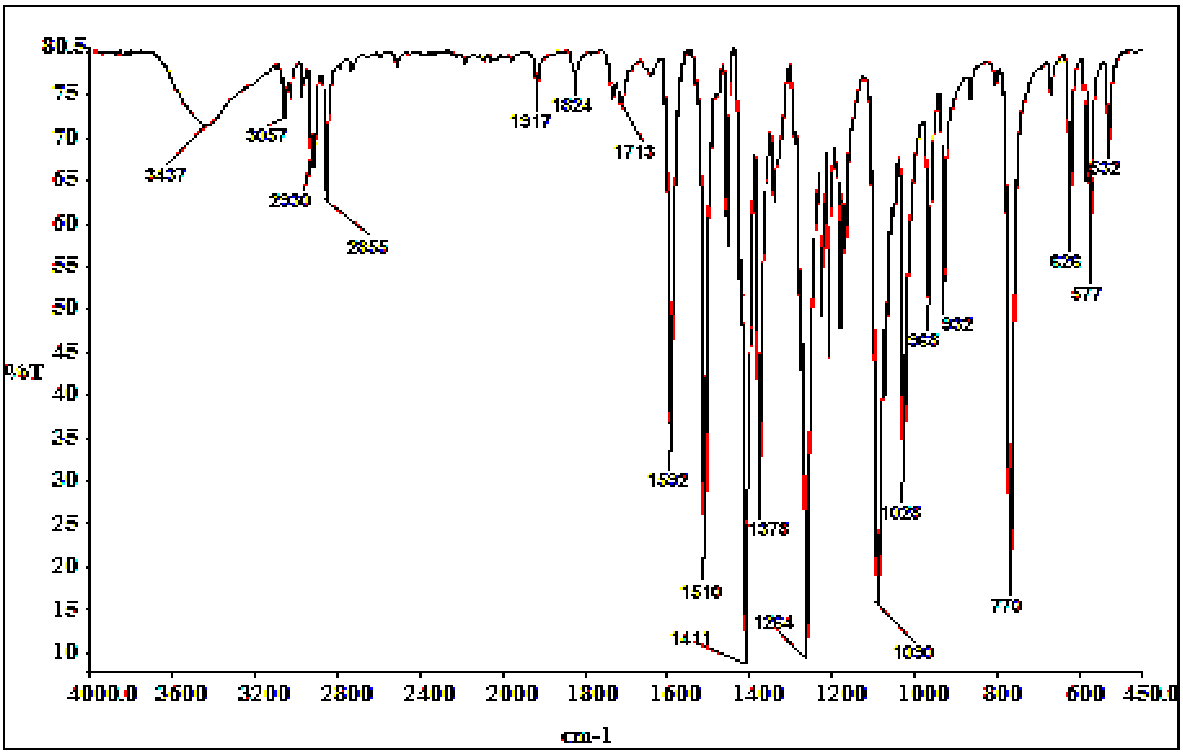

^{1}H NMR of compound 4

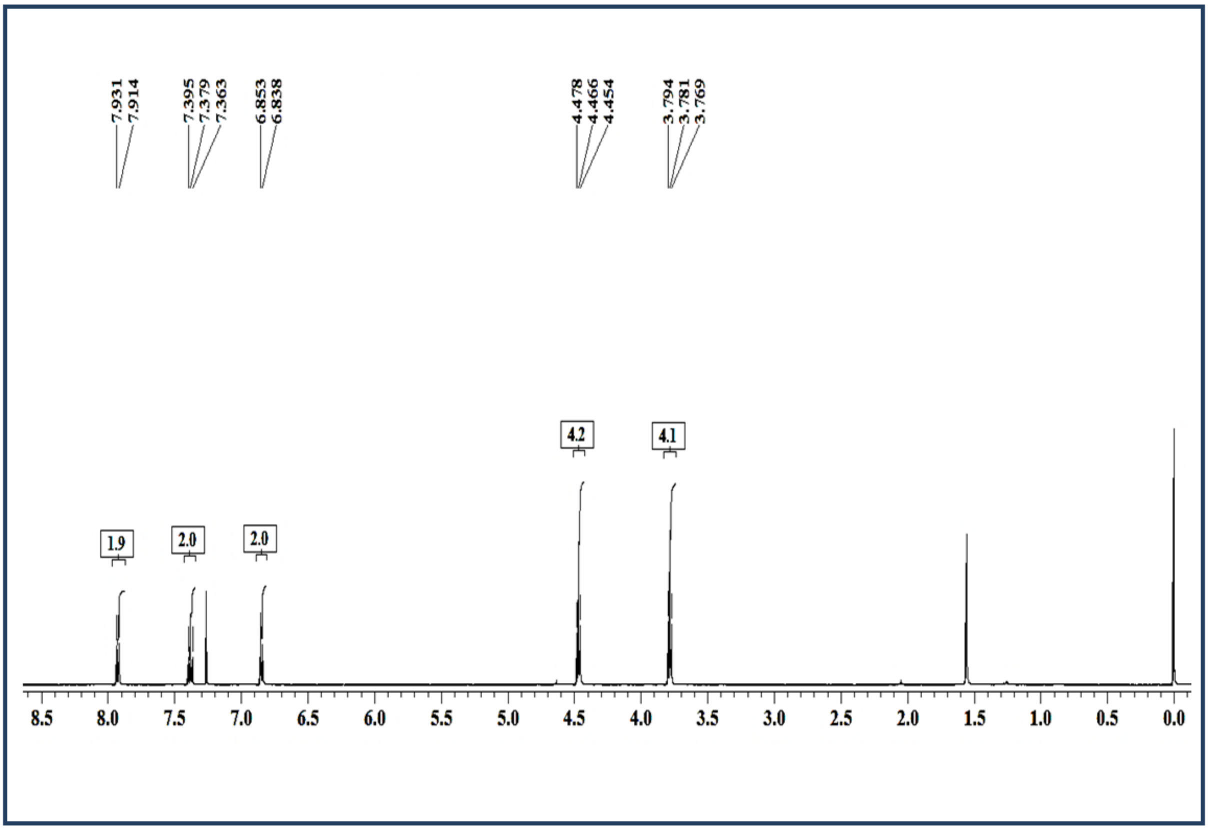

^{13}C NMR of compound 4

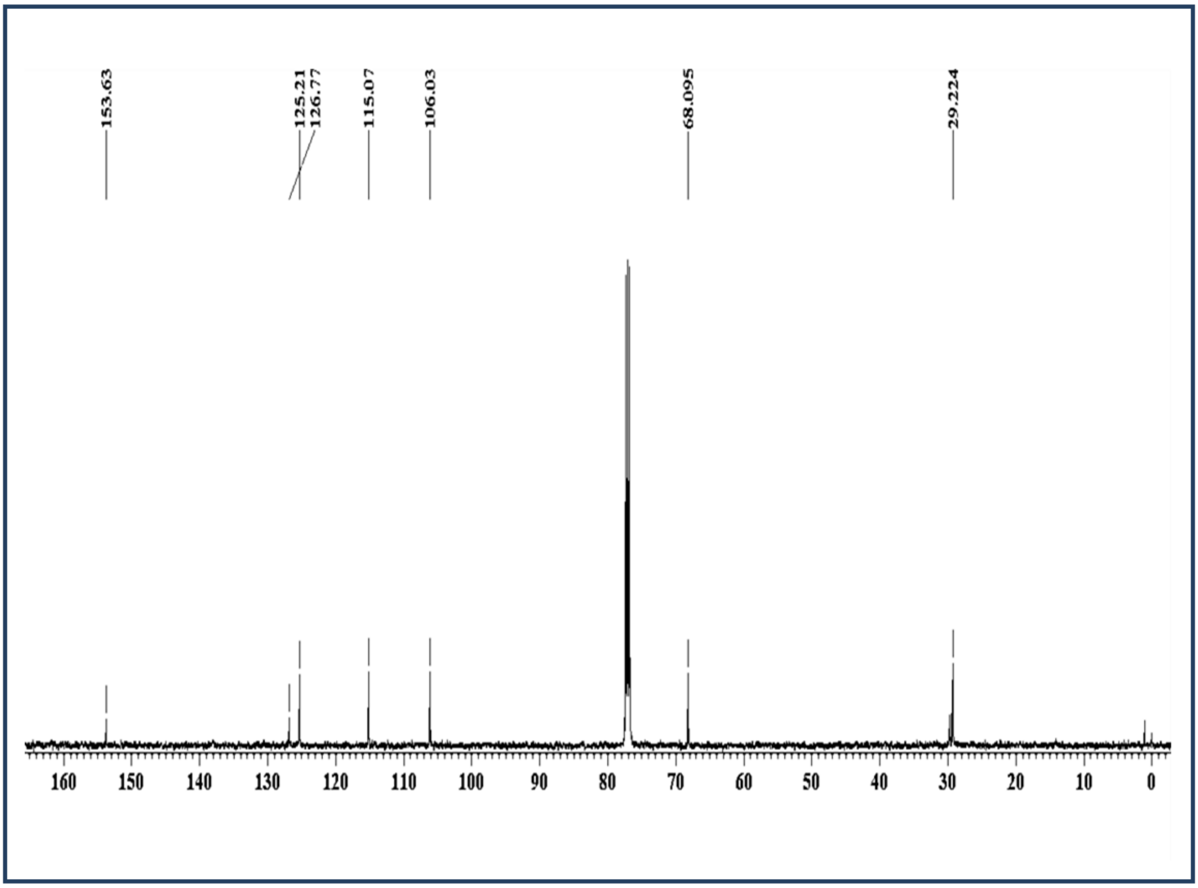

FT-IR of compound 5

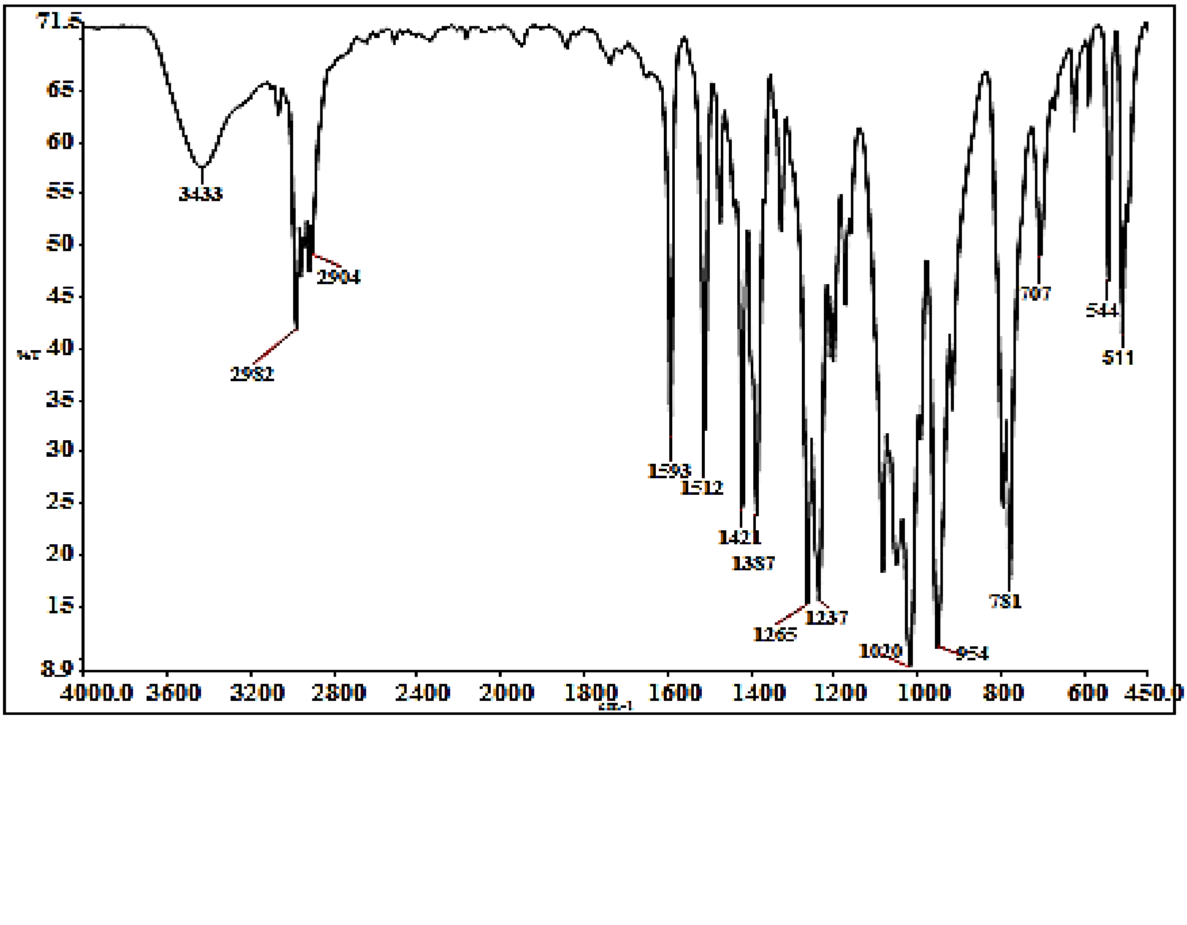

^{1}H NMR of compound 5

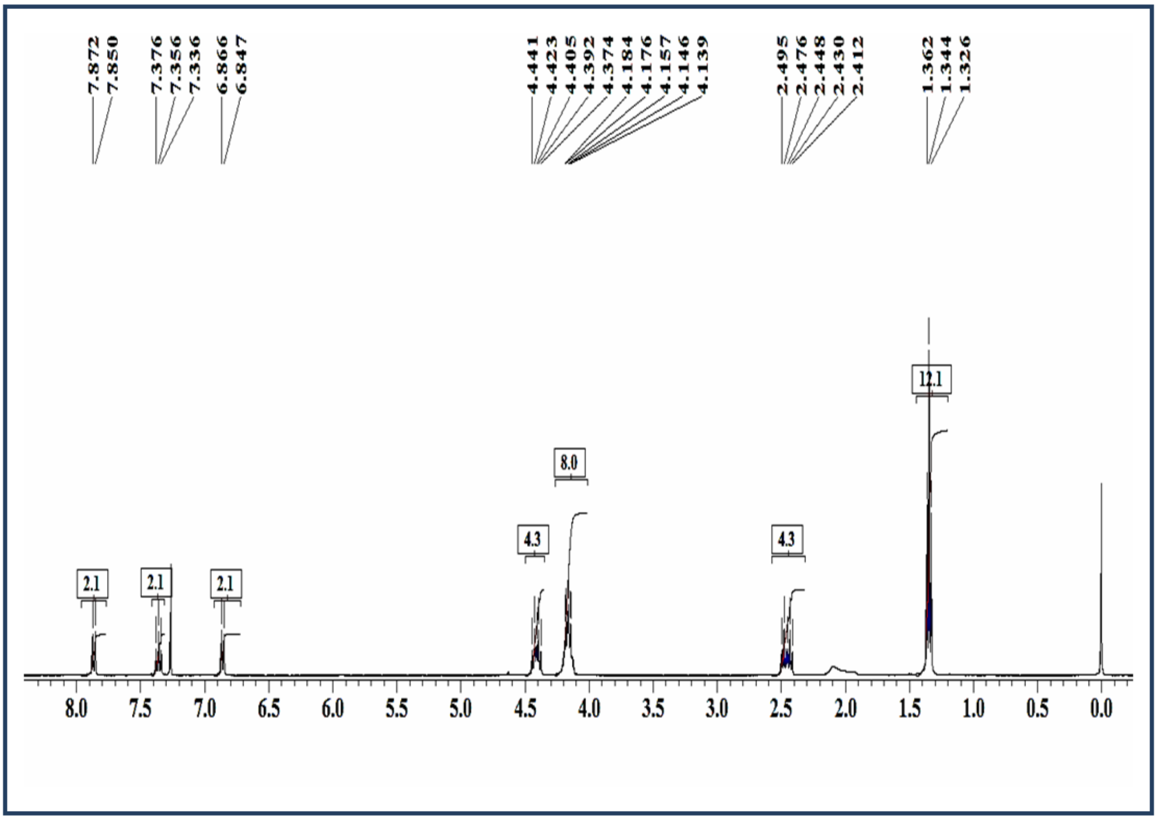

^{13}C NMR of compound 5

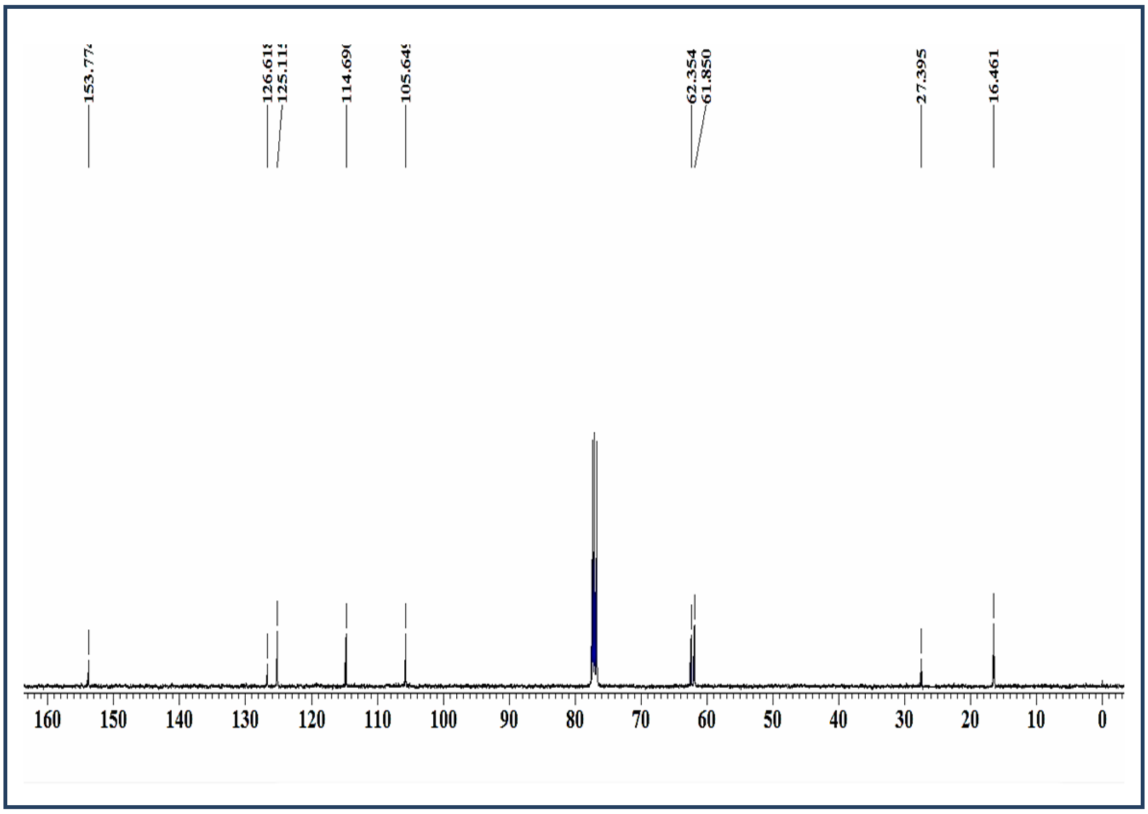

^{31}P NMR of compound 5

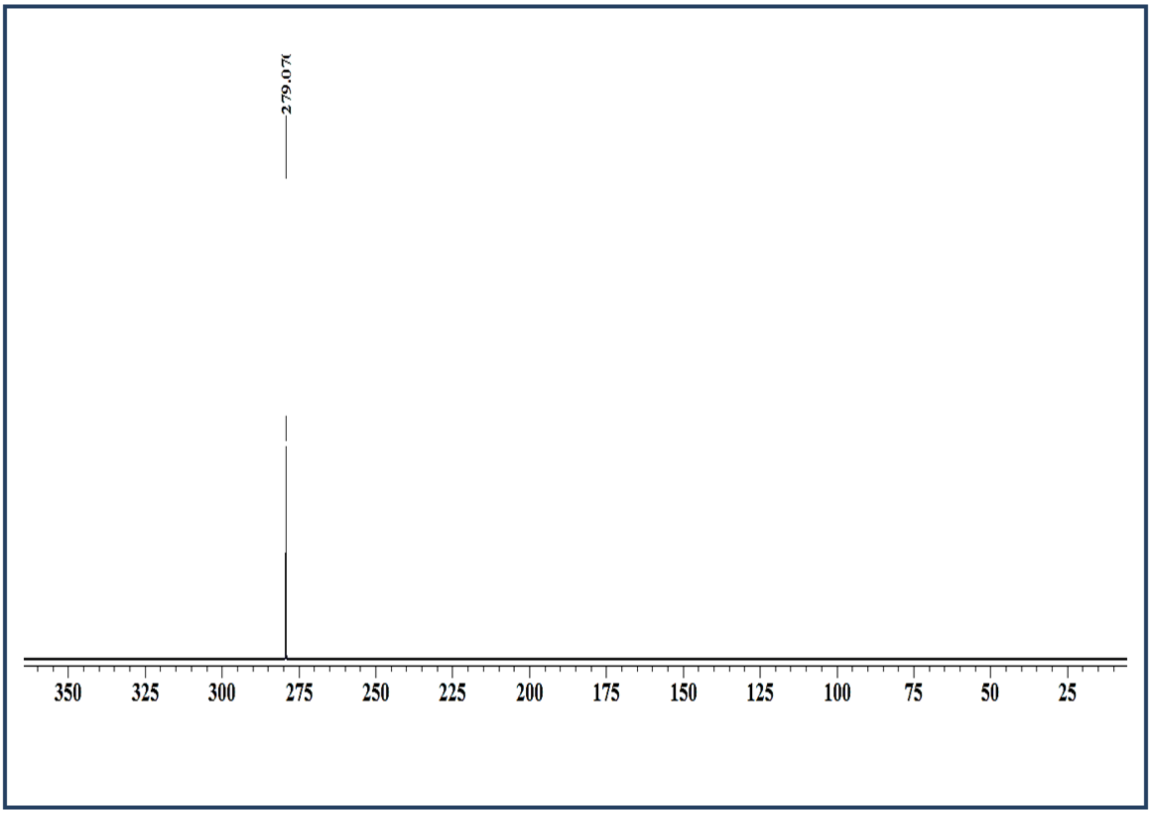

ESI-HRMS of compound 5

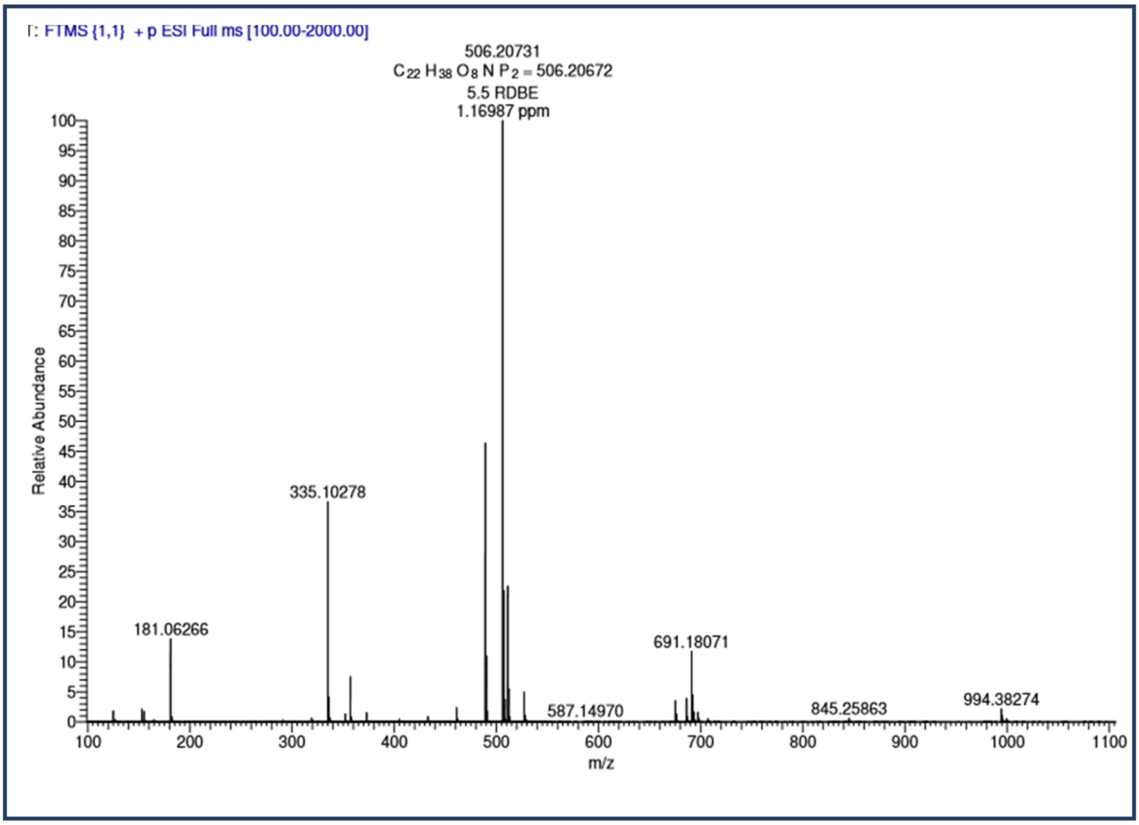

FT-IR of compound 2

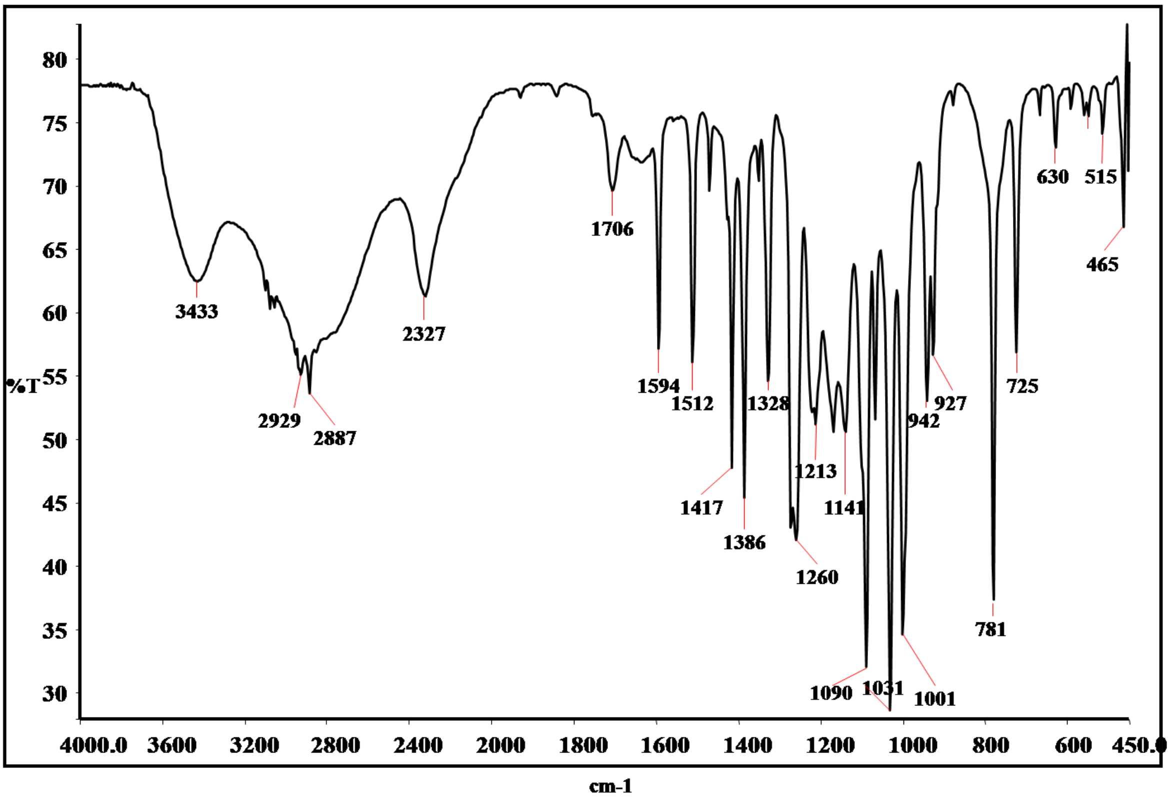

^{1}H NMR of compound 2

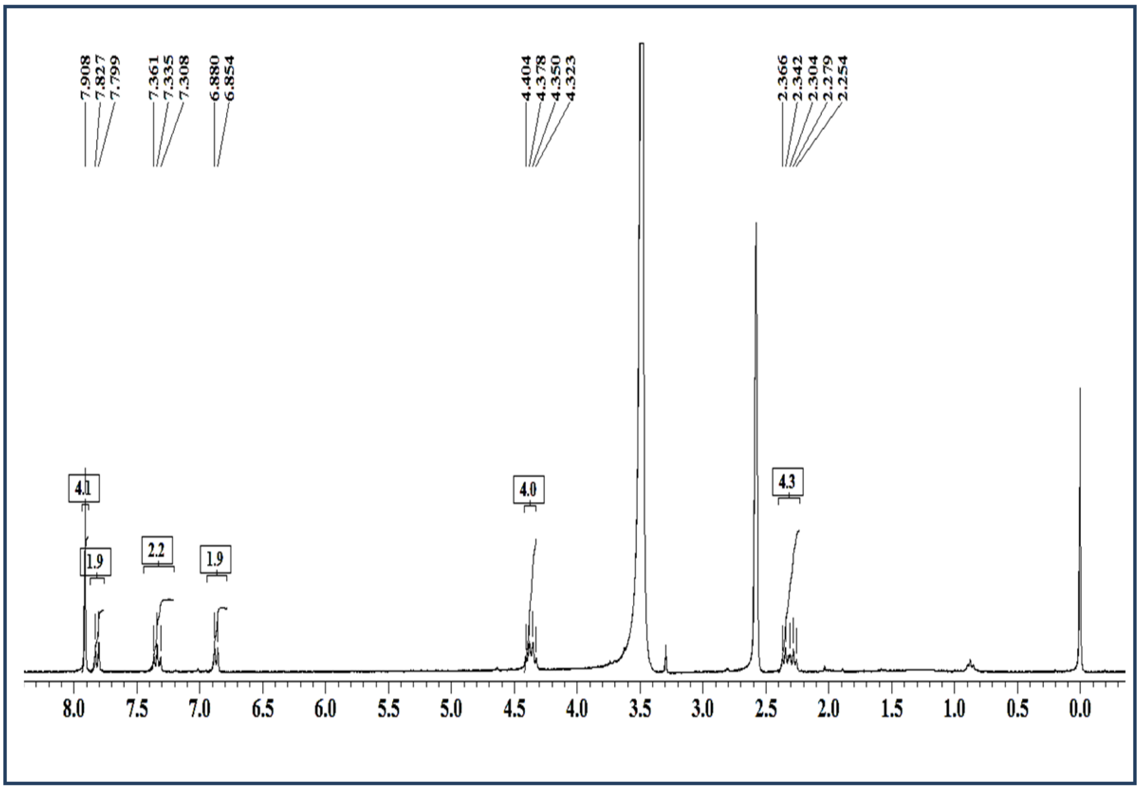

^{13}C NMR of compound 2

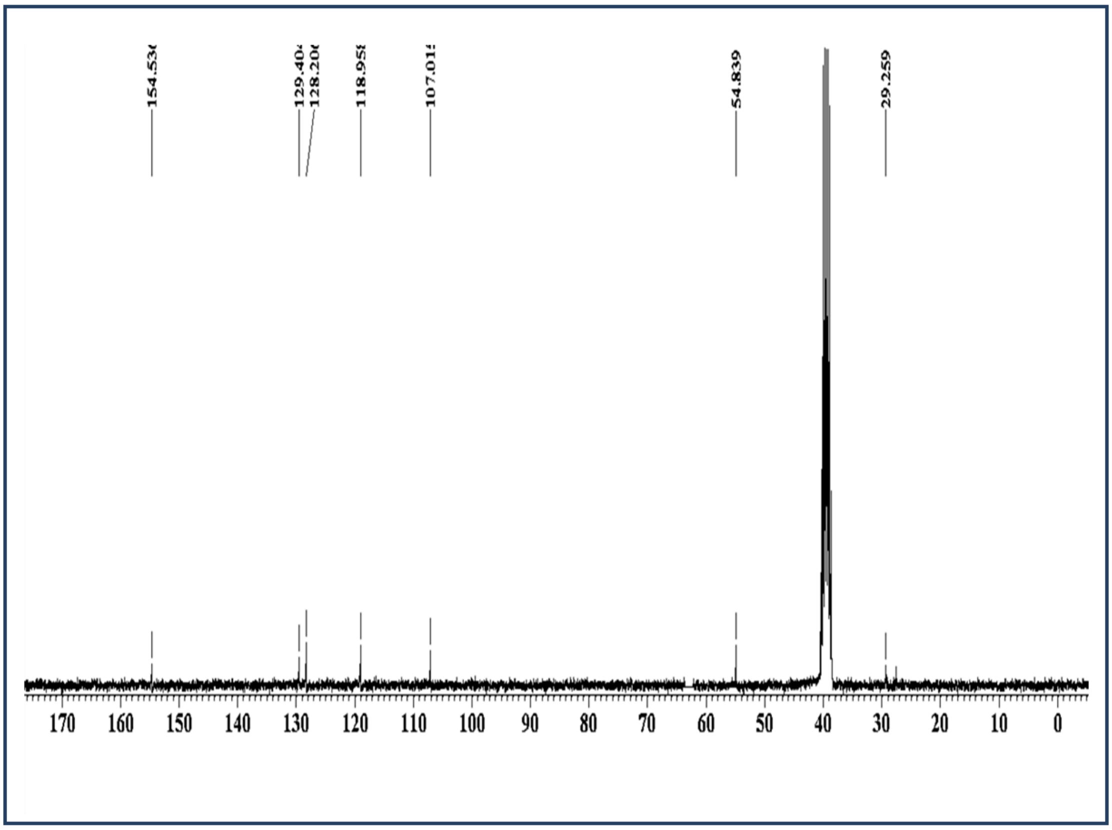

^{31}P NMR of compound 2

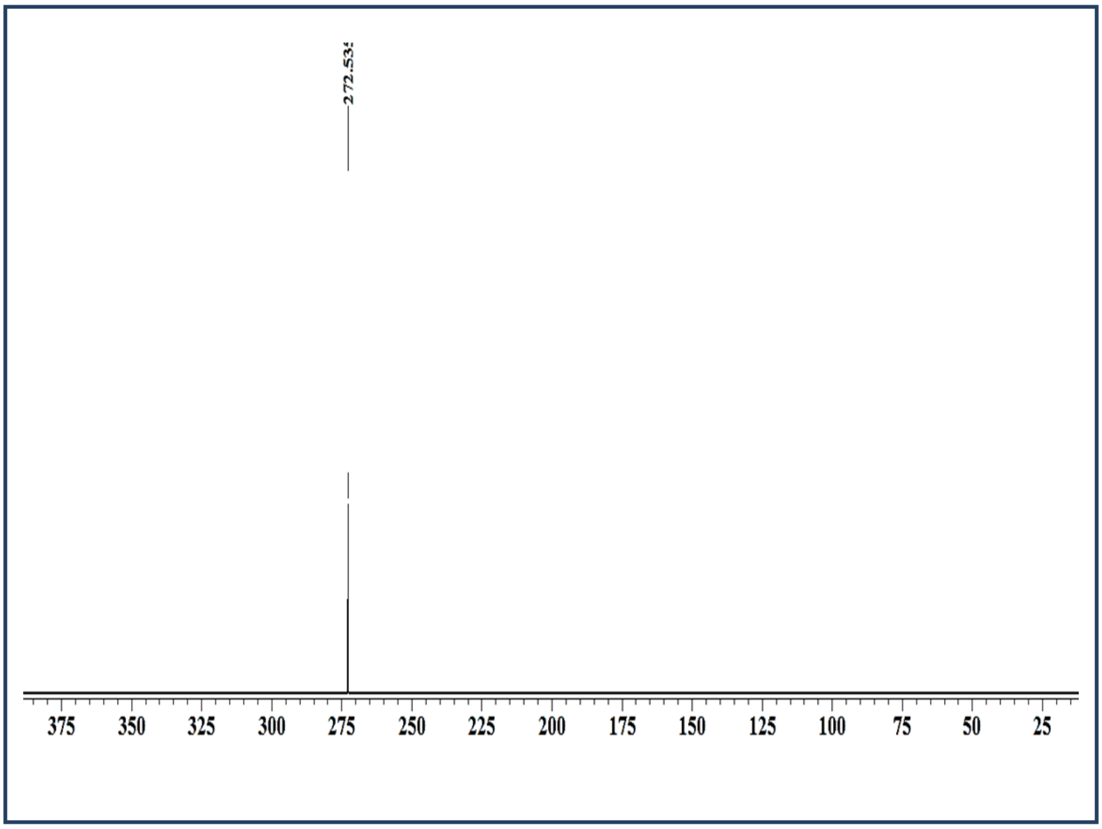

ESI-HRMS of compound 2

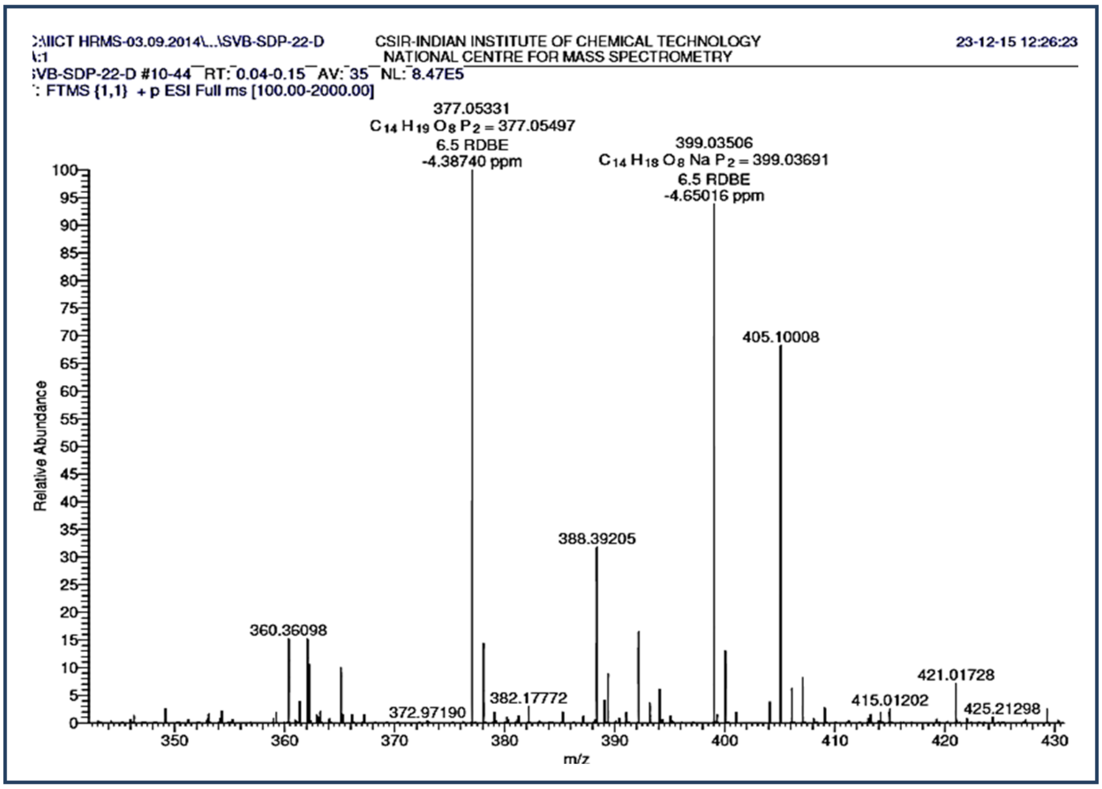

FT-IR of compound 6

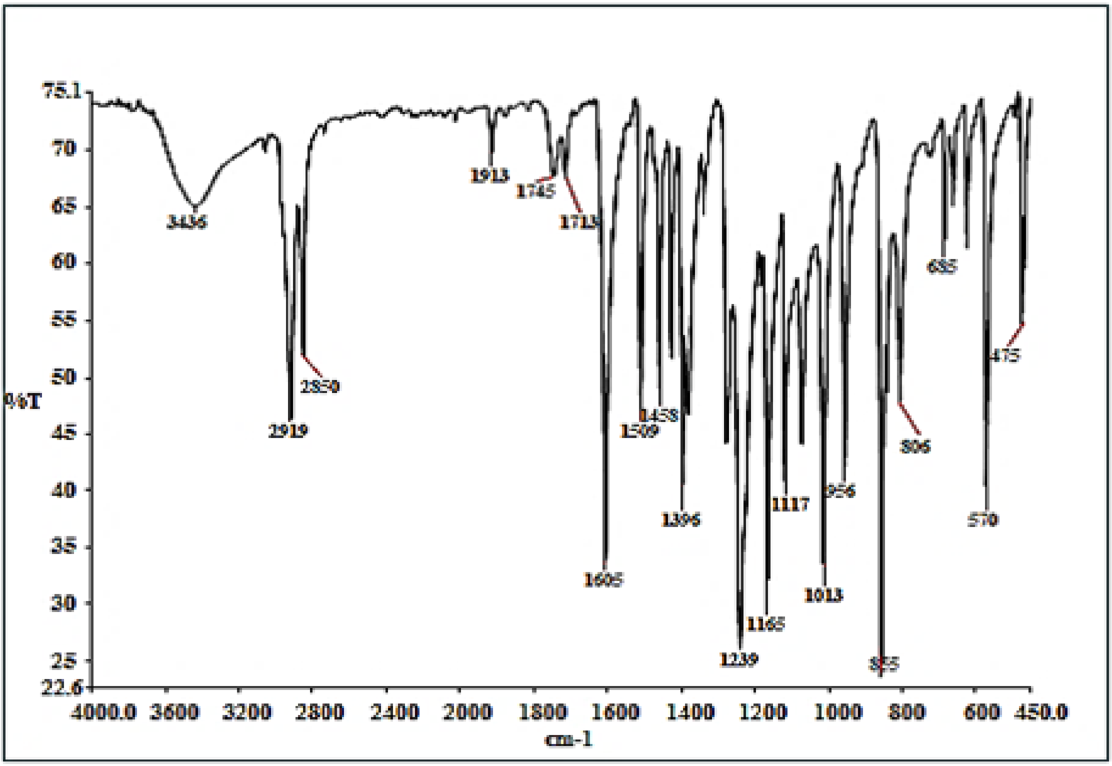

^{1}H NMR of compound 6

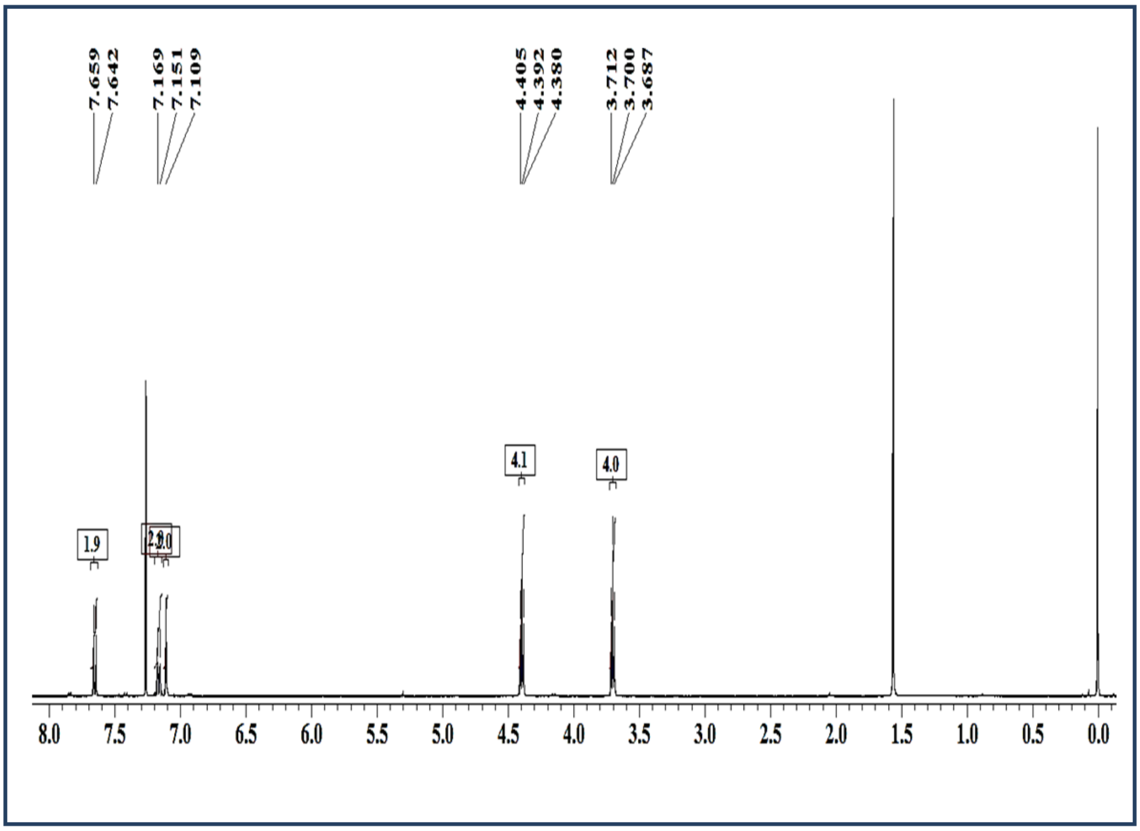

^{13}C NMR of compound 6

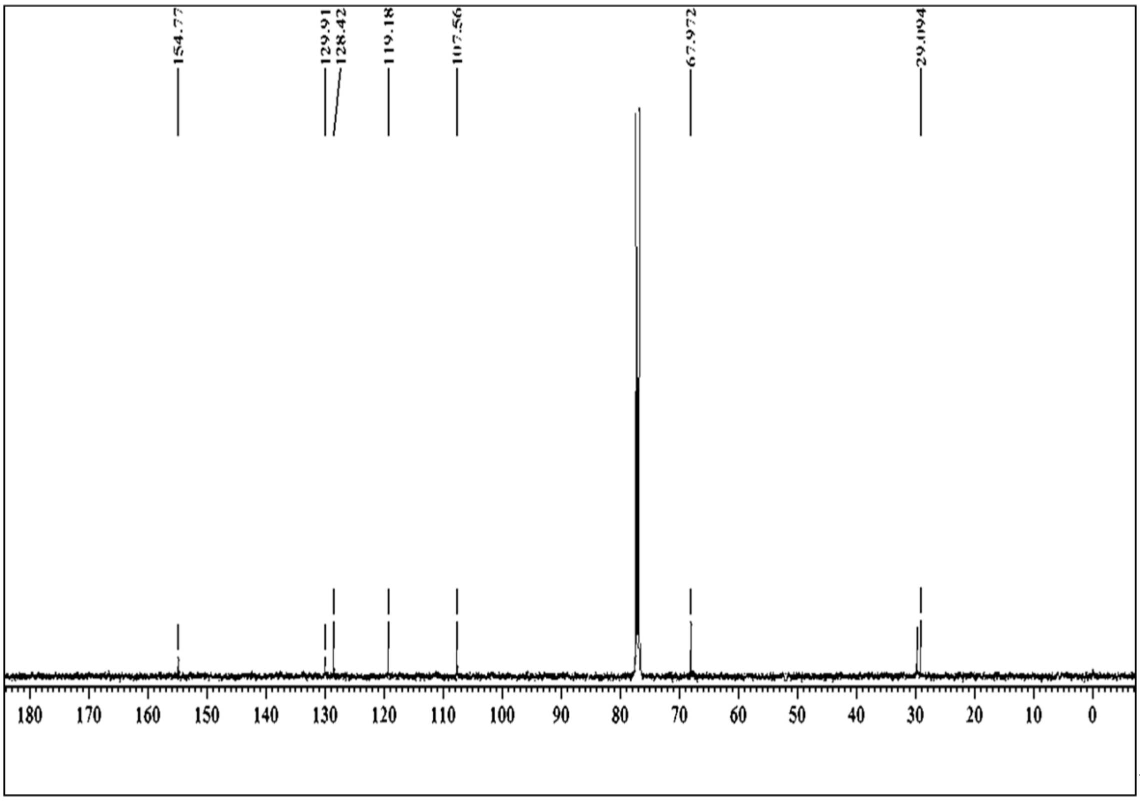

FT-IR of compound 7

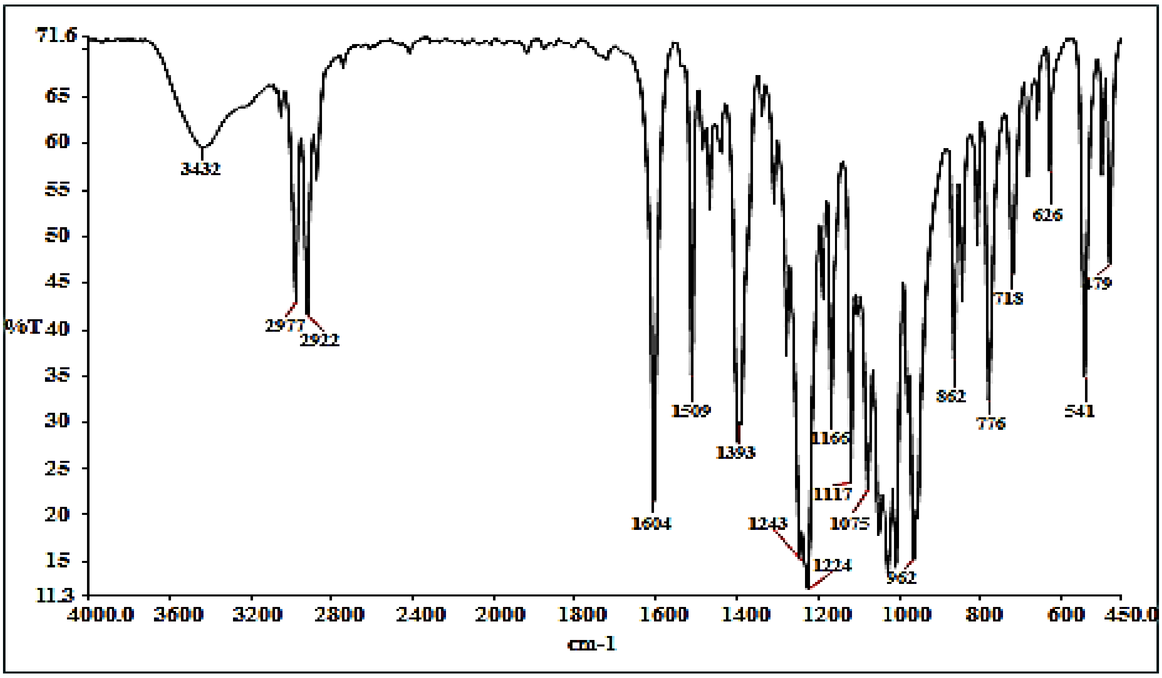

^{1}**H NMR of compound 7**

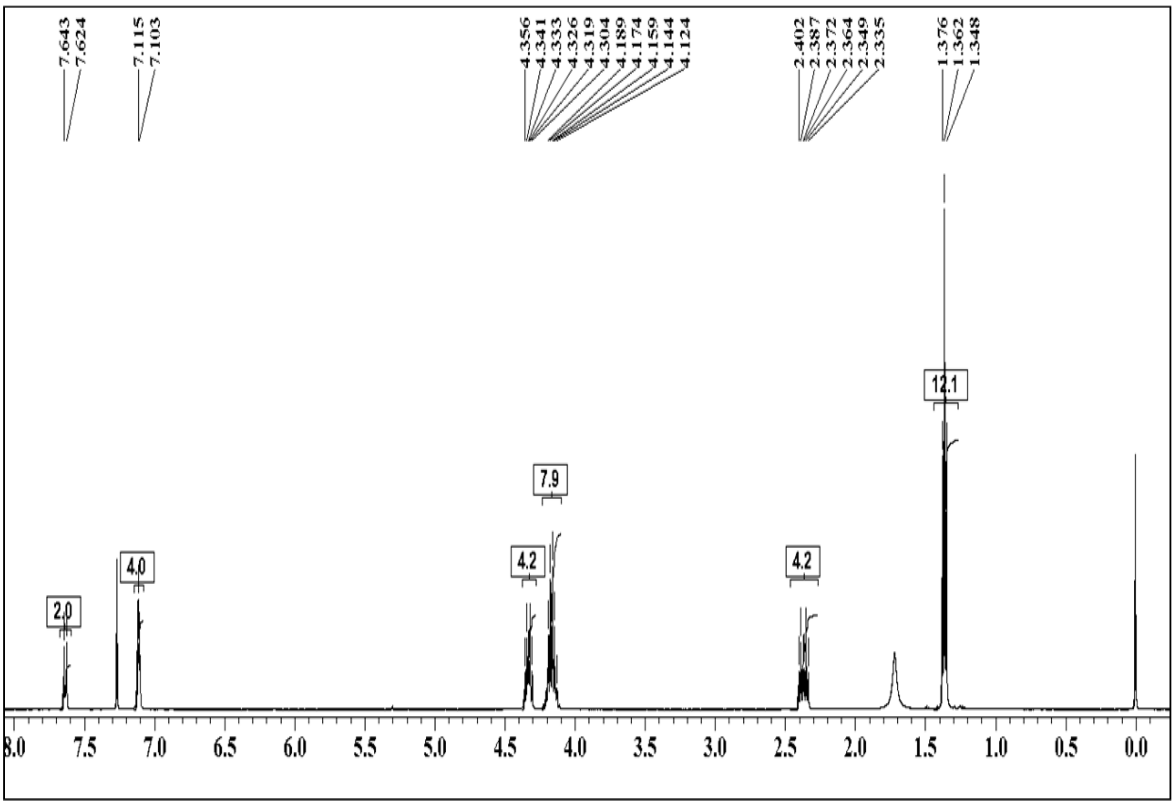

^{13}C NMR of compound 7

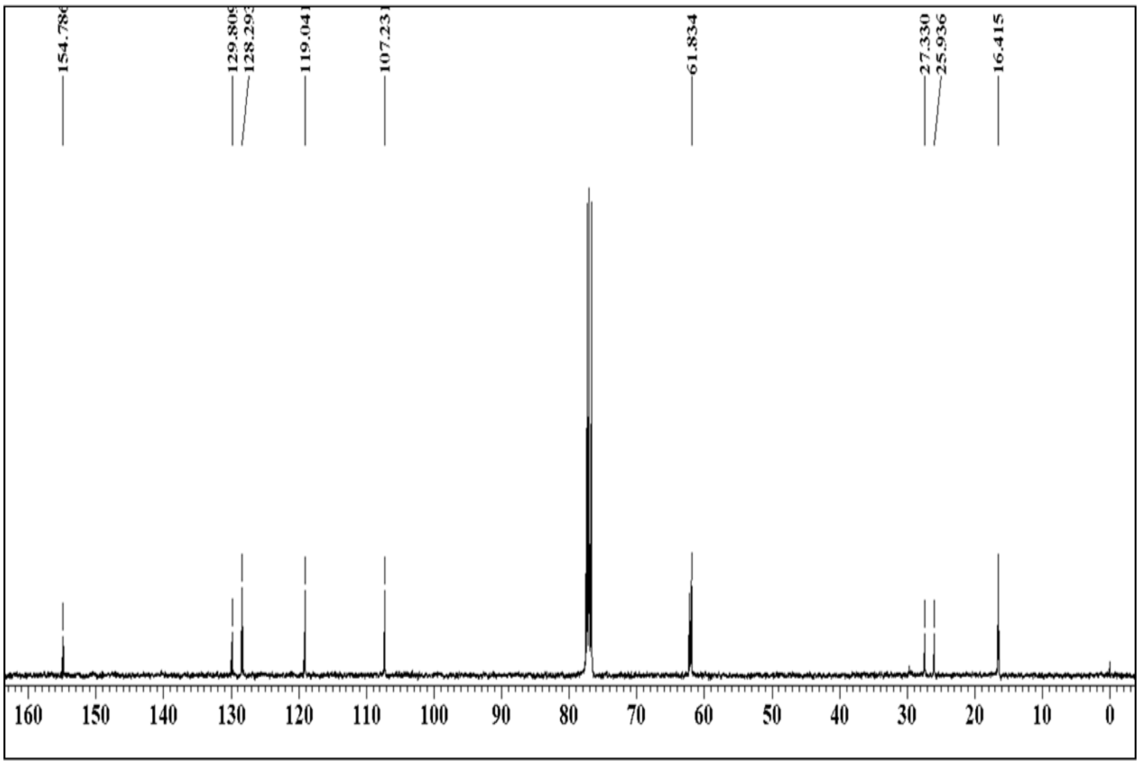

^{31}P NMR of compound 7

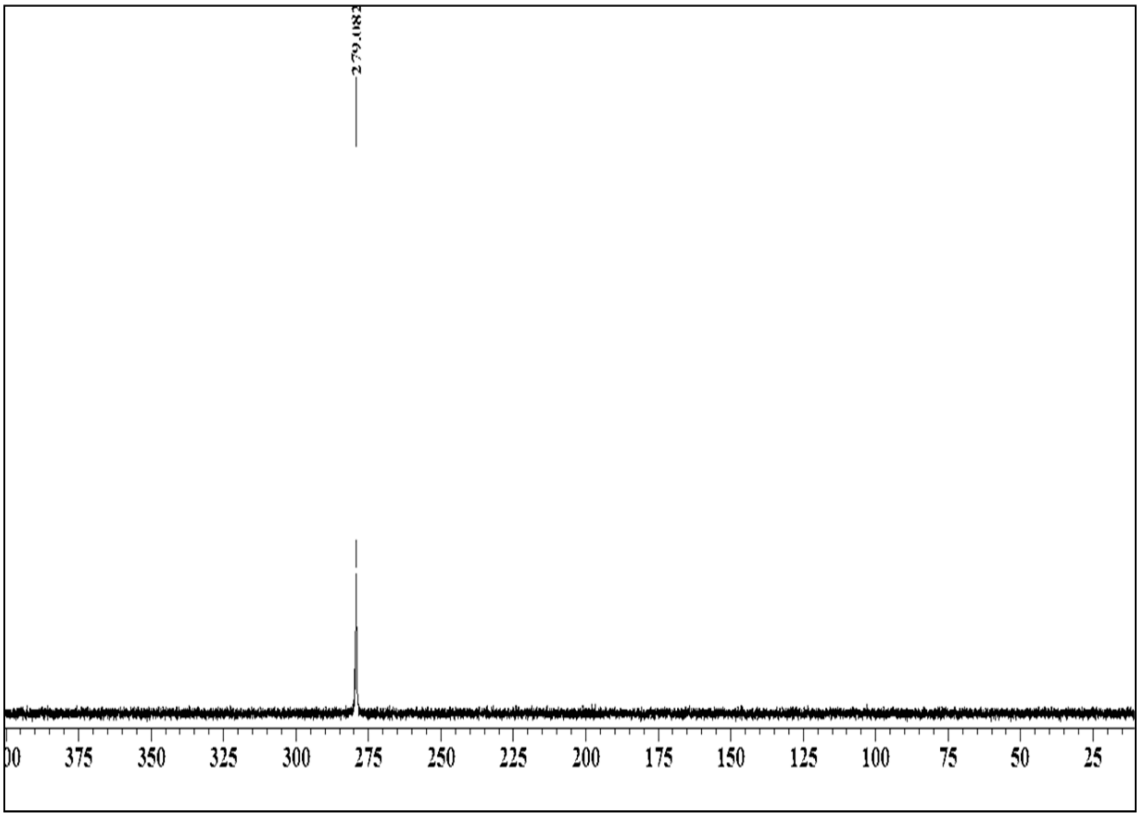

ESI-HRMS of compound 7

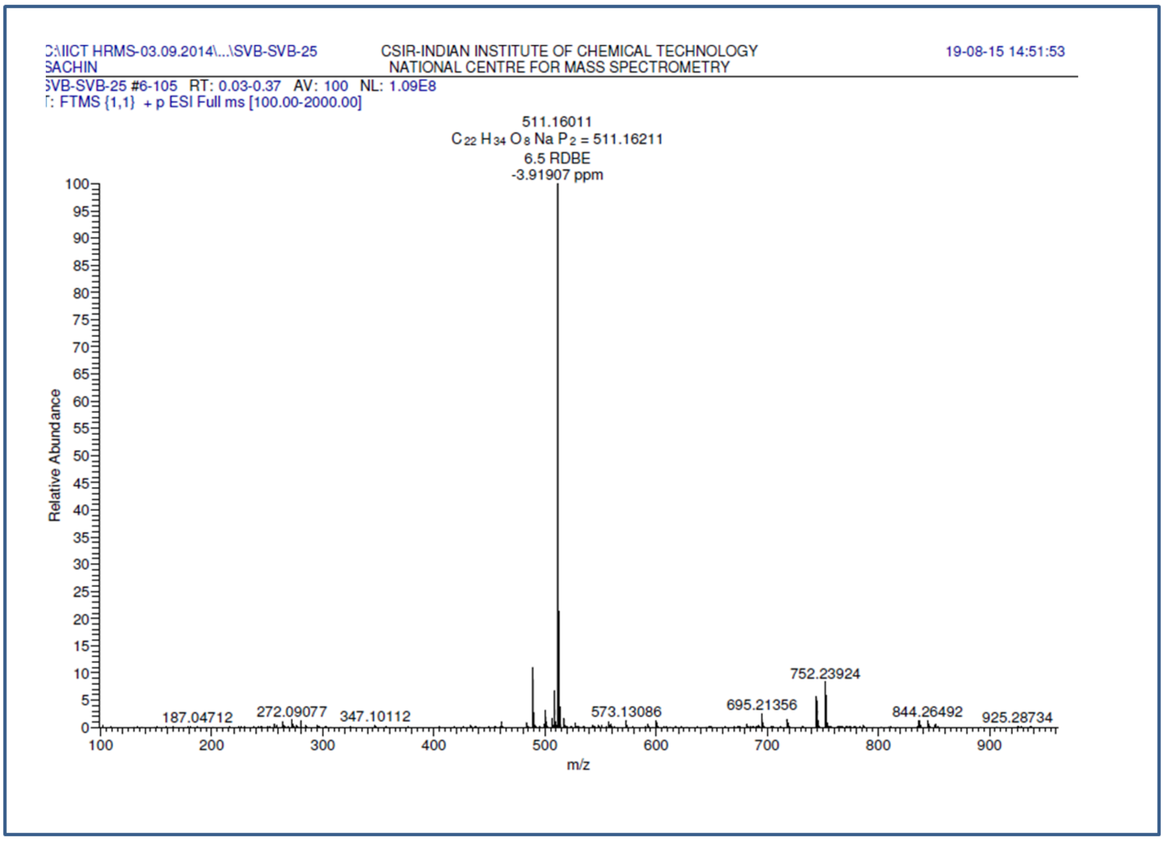

FT-IR of compound 3

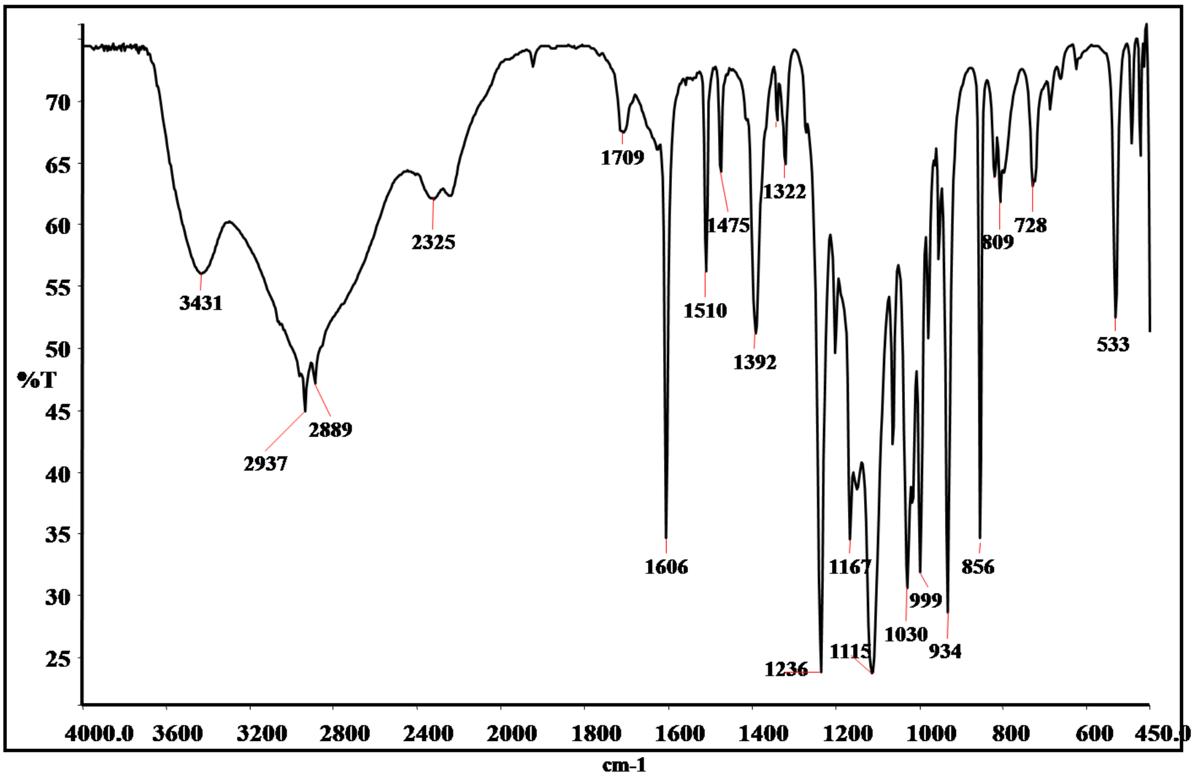

^{1}H NMR of compound 3

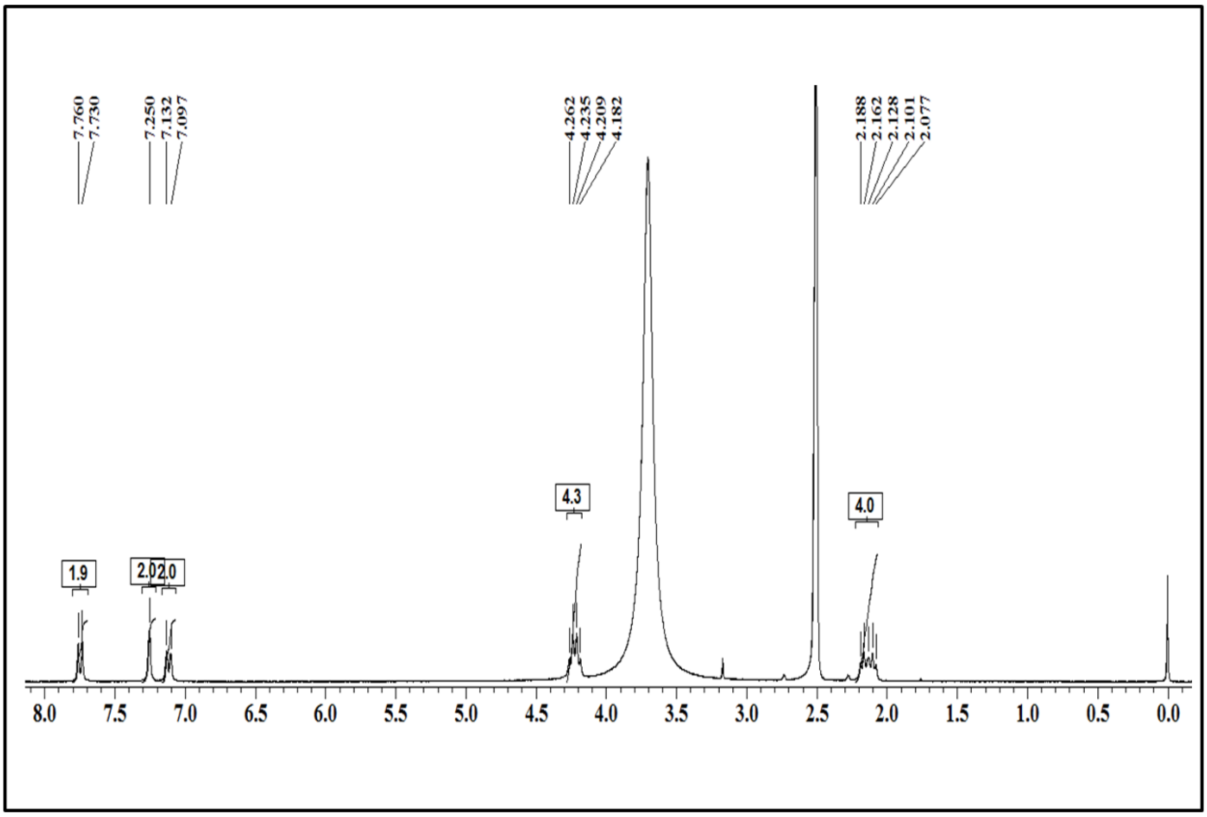

^{13}C NMR of compound 3

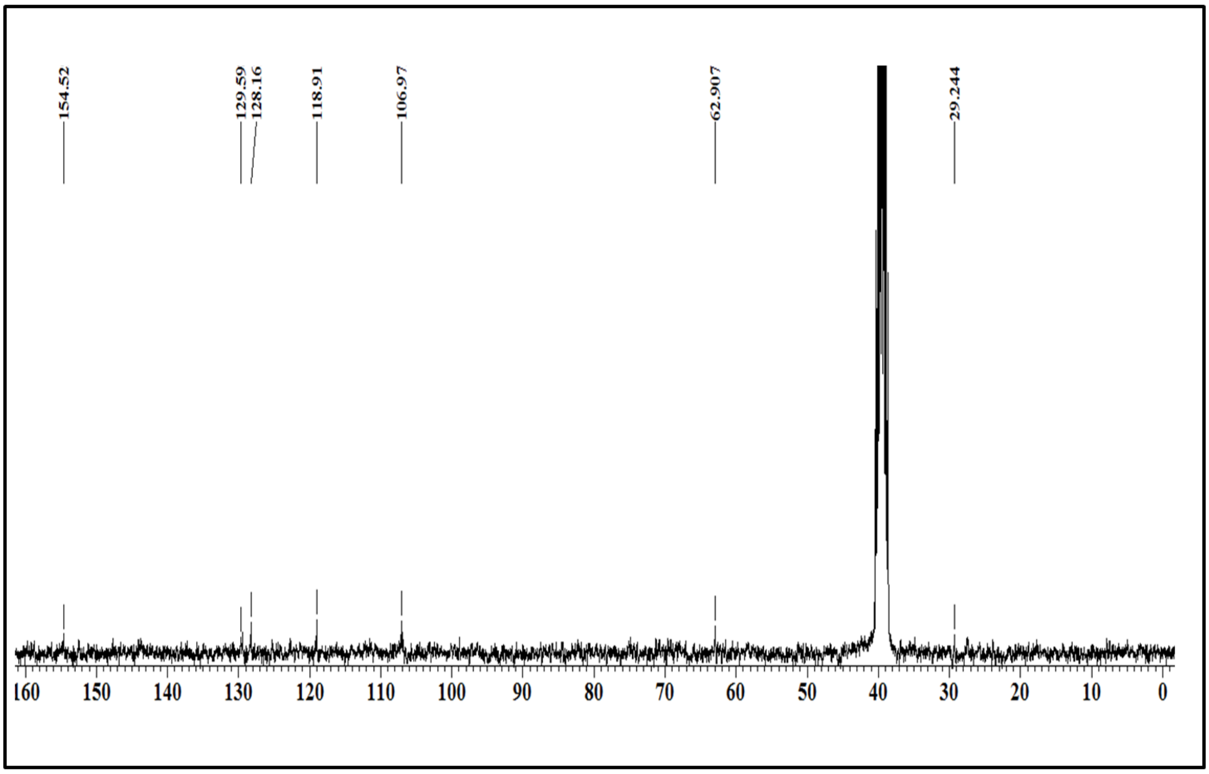

^{31}P NMR of compound 3

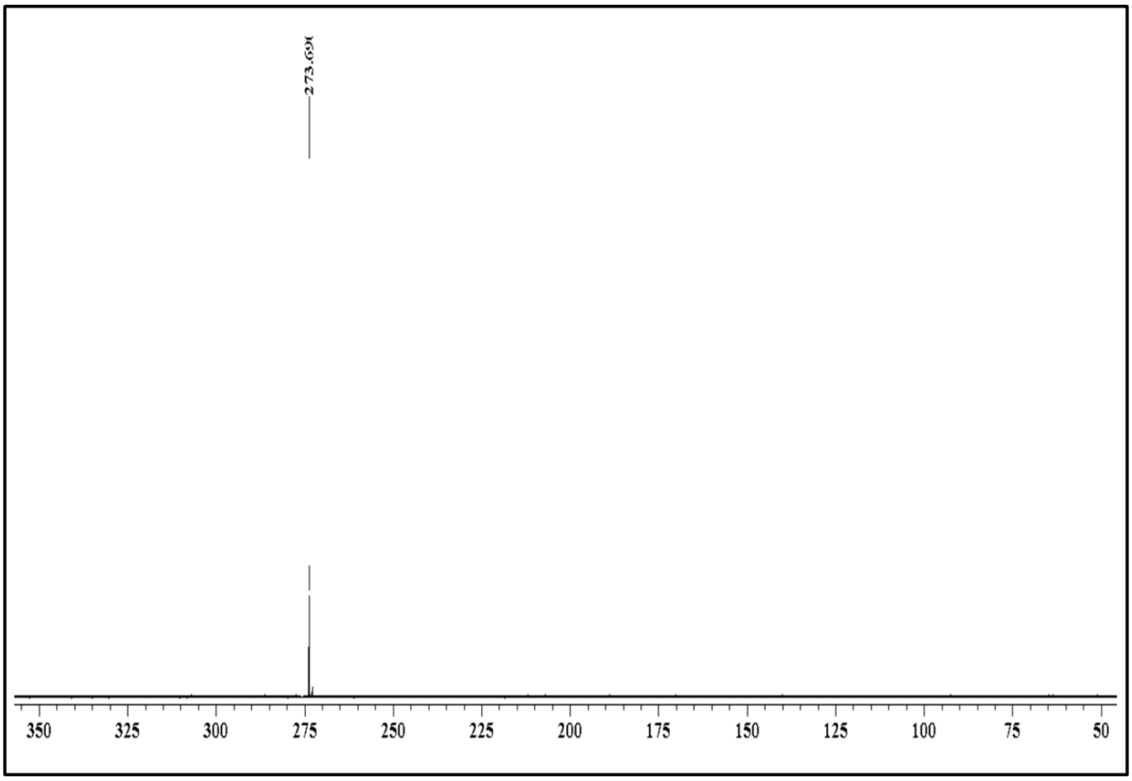

ESI-HRMS of compound 3

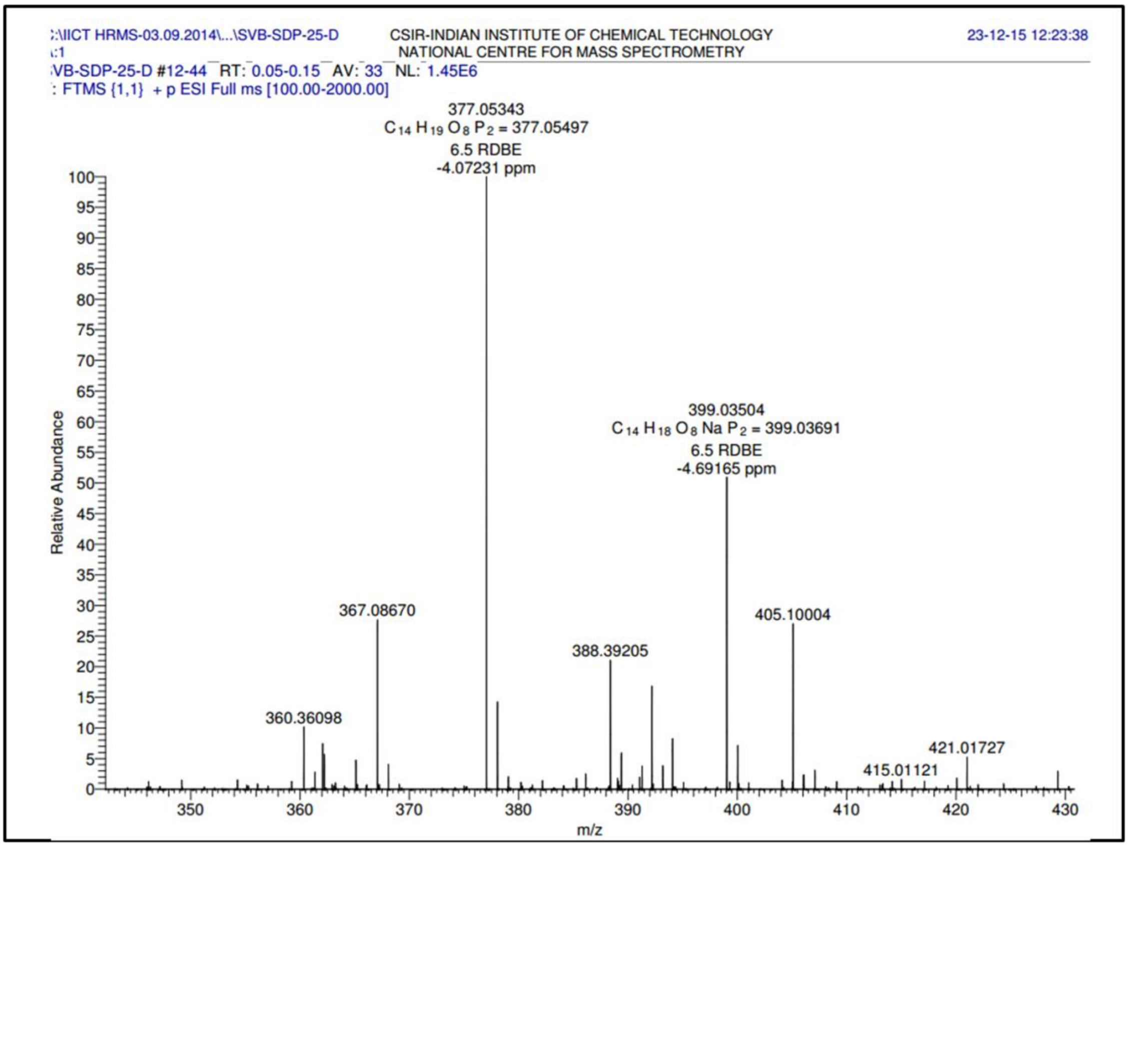

Reference:

S1 M. B. Avinash, E. Verheggen, C. Schmuck and T. Govindaraju, *Angew. Chem., Int. Ed*. 2012, **51**, 10324-10328.
